# The Potency of Essential Oils in Combating Stored-Product Pests: From Nature to Nemesis

**DOI:** 10.3390/plants14020192

**Published:** 2025-01-11

**Authors:** Nickolas G. Kavallieratos, Nikoleta Eleftheriadou, Constantin S. Filintas, Maria C. Boukouvala, Demeter Lorentha S. Gidari, Anna Skourti, Dionysios Ntinokas, Marta Ferrati, Eleonora Spinozzi, Riccardo Petrelli, Filippo Maggi

**Affiliations:** 1 Laboratory of Agricultural Zoology and Entomology, Department of Crop Science, Agricultural University of Athens, 75 Iera Odos Str., 11855 Athens, Greece; nikolelef@aua.gr (N.E.); p1172219@aua.gr (C.S.F.); mbouk@aua.gr (M.C.B.); dlgidari@aua.gr (D.L.S.G.); annaskourti@aua.gr (A.S.); dntinokas@aua.gr (D.N.); 2 Chemistry Interdisciplinary Project (ChIP) Research Center, School of Pharmacy, University of Camerino, Via Madonna delle Carceri, 62032 Camerino, Italy; marta.ferrati@unicam.it (M.F.); eleonora.spinozzi@unicam.it (E.S.); riccardo.petrelli@unicam.it (R.P.); filippo.maggi@unicam.it (F.M.)

**Keywords:** integrated pest management, eco-friendly insecticides, pest control, storage pests, mortality, adults, larvae

## Abstract

*Sitophilus oryzae*, *Tribolium castaneum*, *Tribolium confusum*, *Oryzaephilus surinamensis*, *Rhyzopertha dominica*, *Tenebrio molitor*, *Trogoderma granarium*, *Acarus siro*, and *Alphitobius diaperinus* represent significant arthropod stored-product pests worldwide. To combat these noxious arthropods, the current study examines the pesticidal effect of essential oils (EOs) derived from four aromatic plants, i.e., *Illicium verum* Hook. F., *Citrus reticulata* Blanco, *Monodora myristica* (Gaertn.) Dunal, and *Xylopia aethiopica* (Dunal) A. Rich. Considering the challenge of pesticide resistance, the current study focuses on assessing the efficacy of these EOs as an eco-friendly alternative to traditional synthetic insecticides. Two EO concentrations (500 and 1000 µL/kg wheat) were applied to different life stages of these pests in the bioassays. Mortality rates were monitored over several days under controlled environmental conditions. The findings demonstrated that *C. reticulata* and *I. verum* EOs had elevated insecticidal effects, especially against larval stages, resulting in 100% mortality in several species. On the contrary, *M. myristica* and *X. aethiopica* EOs showed less overall efficacy despite their potency against some pests. Both *I. verum* and *C. reticulata* EOs outperformed the positive control, pirimiphos-methyl, in several assays. The results of the current study highlight the potential of several EOs as effective alternatives in reducing synthetic pesticide use for integrated pest control management.

## 1. Introduction

The bostrychid *Rhyzopertha dominica* (F.), the tenbrionids *Alphitobius diaperinus* (Panzer), *Tribolium castaneum* (Herbst), *Tribolium confusum* Jacquelin du Val, and *Tenebrio molitor* L., the curculionid *Sitophilus oryzae* (L.), the dermestid *Trogoderma granarium* Everts, the sylvanid *Oryzaephilus surinamensis* (L.), and the acarid *Acarus siro* L., are major pests of stored commodities that are spread globally [1,2]. These species cause serious infestations and degradation in storage units containing cereal, grain by-products, pulses, fruits, nuts, vegetables, and even animal-derived products [3]. In addition to the qualitative and quantitative damages caused by these arthropod pests, their activity in different agricultural settings may lead to the accumulation of contaminants, like feces, body fragments, and secretions [4]. Several of these pests are linked with human, pet, and farm animal health hazards such as allergies and diseases [5,6,7]. The constant global distribution of stored-product arthropod populations through international commerce, along with occurring and documented pesticide resistance [8,9,10], necessitates further research and development of novel pest control measures [11,12,13].

*Illicium verum* Hook. f. (Austrobaileyales: Schisandraceae) is an aromatic evergreen tree known for its reddish, star-shaped fruits and blossoms. It is predominantly cultivated in Southern Asia, particularly in China and Vietnam. The fruit of the tree, commonly known as star anise, is widely used as a cooking spice and has a significant role in traditional Chinese medicine for its therapeutic properties [14]. *Illicium verum* is widely utilized in herbal medicine, due to the effectiveness of its bioactive constituents, particularly (*E*)-anethole [15]. Recent research highlights that the fruit of *I. verum* possesses important antioxidant, antimicrobial, and anti-inflammatory activities [16,17].

*Xylopia aethiopica* (Dunal) A. Rich. (Magnoliales: Annonaceae) is extensively distributed throughout Africa, particularly in the Western and central regions [18]. It is an evergreen tree, often reaching heights of over 20 m, producing fruits that are slightly hooked cylindrical pods, measuring 2 to 3 mm in width [18]. The plant holds cultural significance in various regions of West Africa due to its medicinal properties and wide usage as a spice. Indeed, many studies have demonstrated its anti-inflammatory, anti-anaphylactic, and antipyretic effects [19,20], including its application for the management of postpartum hemorrhage and contractions [21]. The fruit extracts and stem bark decoctions are traditionally used to cure conditions such as biliousness, bronchitis, and dysentery [22,23]. In addition, it has been proven to be a potent antimicrobial agent against serious infectious microorganisms, like *Bacillus subtilis*, *Staphylococcus aureus*, and *Candida albicans* [19,24].

*Monodora myristica* (Gaertn.) Dunal (Magnoliales: Annonaceae), commonly known as calabash nutmeg, is a tropical perennial species indigenous to West Africa [25]. This tree typically reaches heights of 15 to 20 m and bears large, glossy leaves [26]. Its distinctive fruit bears a hard, woody shell resembling a calabash, housing seeds that emit a rich, nutmeg-like fragrance [27]. For a long time, these aromatic seeds have been used as a culinary spice and in traditional folk medicine, while recent research focused on their pharmacological potential [28]. The bioactive compounds found in the seeds and stem bark exhibit strong antioxidant and anti-inflammatory properties [29,30]. The stem bark is often utilized as a remedy for ailments such as stomach aches, hemorrhoids, fever, and eye ailments [31]. Additionally, many studies demonstrated its cytotoxicity against cancer cells [32] as well as its antibacterial [33] and antiparasitic effects [34].

*Citrus reticulata* Blanco (Sapindales: Rutaceae), commonly known as mandarin, represents a prominent and economically significant plant worldwide [35]. Originating in Southeast Asia, this fruit-bearing evergreen tree has become widely cultivated, due to its flavorful and aromatic fruits [36,37]. Unlike common oranges, mandarins can range from oblate to spherical, depending on the variety [38]. Moreover, *Citrus* fruit peels are rich in phenolics and flavonoids, responsible for many of their bioactivities, generating scientific interest [39]. Previous studies have focused on their antioxidant [40], anti-inflammatory [41], potential anti-cancer [36], antimicrobial [37], and anti-diabetic effects [42].

Given the limited knowledge on the EOs from *I. verum*, *X. aethiopica*, *M. myristica*, and *C. reticulata* as grain protectants [43,44], the current study aimed at investigating their potential as bio-insecticides against key primary and secondary pests. Given the widespread problem of pesticide resistance and the growing need for sustainable pest management strategies, this research effort aimed to assess the pesticidal potential of these EOs at varying concentrations against eight economically important arthropod pest species. In addition, the present study evaluated the comparative effectiveness of the tested EOs in relation to the conventional pesticide pirimiphos-methyl in reducing pest populations.

## 2. Results

### 2.1. EO Chemical Compositions

The GC-MS analysis of *M. myristica*, *X. aethiopica*, *I. verum*, and *C. reticulata* EOs led to the identification of the 92.9, 99.6, 99.7, and 99.3% of the total composition, respectively (Table 1). As previously reported in the work of Wandjou et al. [45], *M. myristica* EO was mainly characterized by monoterpene hydrocarbons, i.e., *p*-cymene (32.8%), *α*-phellandrene (32.3%), *α*-pinene (7.6%), limonene (4.4%), and myrcene (4.3%), while *X. aethiopica* EO was dominated by monoterpene hydrocarbons, oxygenated monoterpenes, and sesquiterpene hydrocarbons with sabinene (26.1%), *β*-pinene (17.4%), germacrene D (9.7%), α-pinene (9.6%), *β*-phellandrene (6.2%), and terpinen-4-ol (6.1%) as main compounds (Table 1). Concerning *I. verum*, its EO was dominated by phenylpropanoids, being (*E*)-anethole the predominant compound (92.6%). On the other hand, *C. reticulata* EO was dominated by monoterpene hydrocarbons, with limonene (97.1%) as the main representative of this chemical class (Table 1).

### 2.2. Effectiveness Against A. diaperinus Adults and Larvae

**Figure 1:**
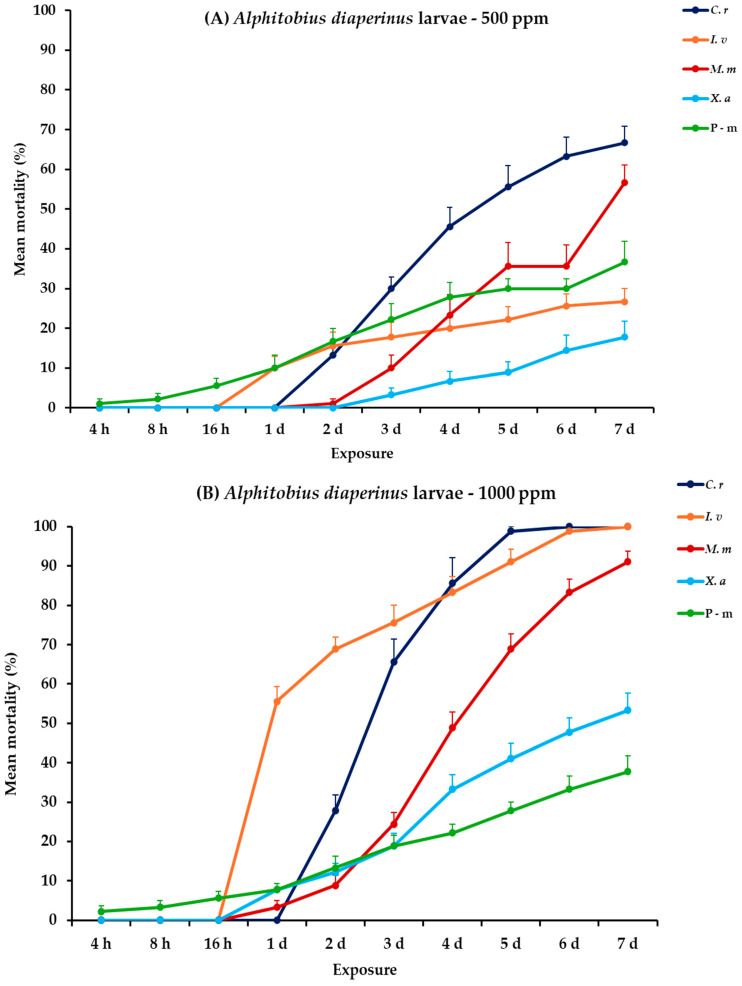

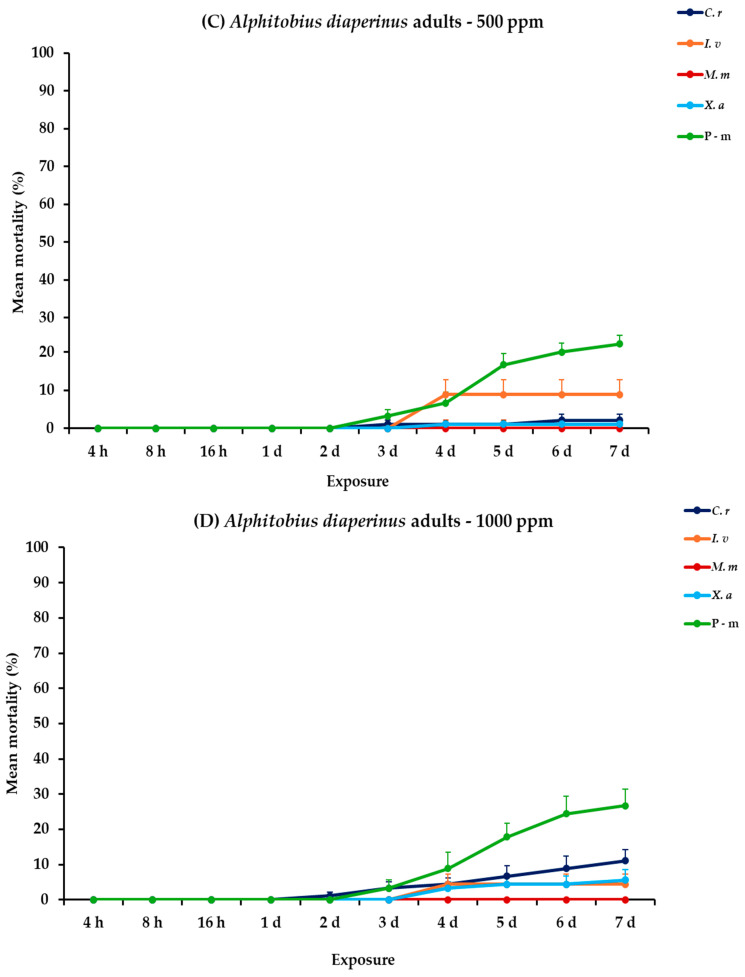
Mean (%) mortality + standard error (SE) of *Alphitobius diaperinus* larvae (**A**,**B**) and adults (**C**,**D**) after 4–16 h and 1–7 days in wheat treated with *C. reticulata*, *I. verum*, *M. myristica*, and *X. aethiopica* (under the abbreviations *C. r*, *I. v*, *M. m*, and *X. a*, respectively) EOs at two concentrations and pirimiphos-methyl (under the abbreviation P-m) as positive control.

All main effects and their interactions were significant between and within exposures for both adult and larval stages of *A. diaperinus*, except the main effect concentration and the three-way interaction between exposure, concentration, and EO for adults (Table 2). *Citrus reticulata* and *I. verum* at the highest concentration demonstrated significant effectiveness against *A. diaperinus* larvae, generally after the fifth day post-exposure, whereas their impact on adults was considerably less pronounced. At 500 ppm, all four EOs exhibited relatively low initial larval mortality (<16%) over a two-day period, with *C. reticulata* and *M. myristica* surpassing 50% mortality on day 5, ultimately reaching 66.7 and 56.7% at 7 days post-exposure. Meanwhile, the positive control, pirimiphos-methyl (P-m), demonstrated a consistent increase with time but peaked at 36.7% on day 7. At the higher concentration (1000 ppm), *C. reticulata* and *I. verum* achieved complete mortality (100%) on day 6 and day 7, respectively. *Monodora myristica* followed with 91.1% mortality by day 7. Conversely, *X. aethiopica* and P-m continued to exhibit elevated efficacy, with *X. aethiopica* reaching 53.3% and P-m exhibiting a similar mortality trend (37.8%). For adult mortality, recorded rates were generally low across all treatments, with *C. reticulata* showing the maximum mortality of 11.1% by day 7 at 1000 ppm (Figure 1, Table S1).

### 2.3. Effectiveness Against T. castaneum Adults and Larvae

**Figure 2:**
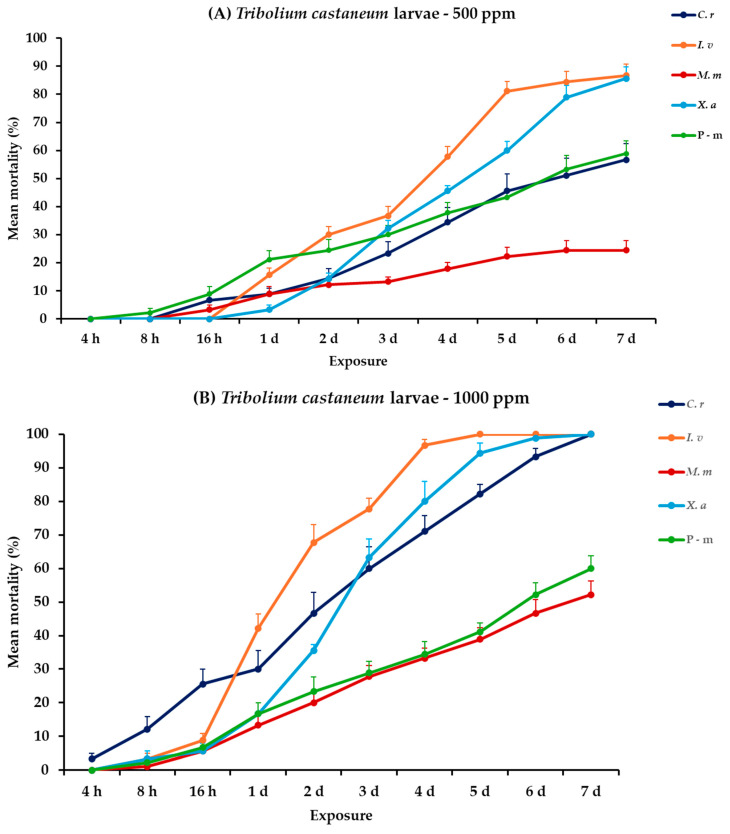

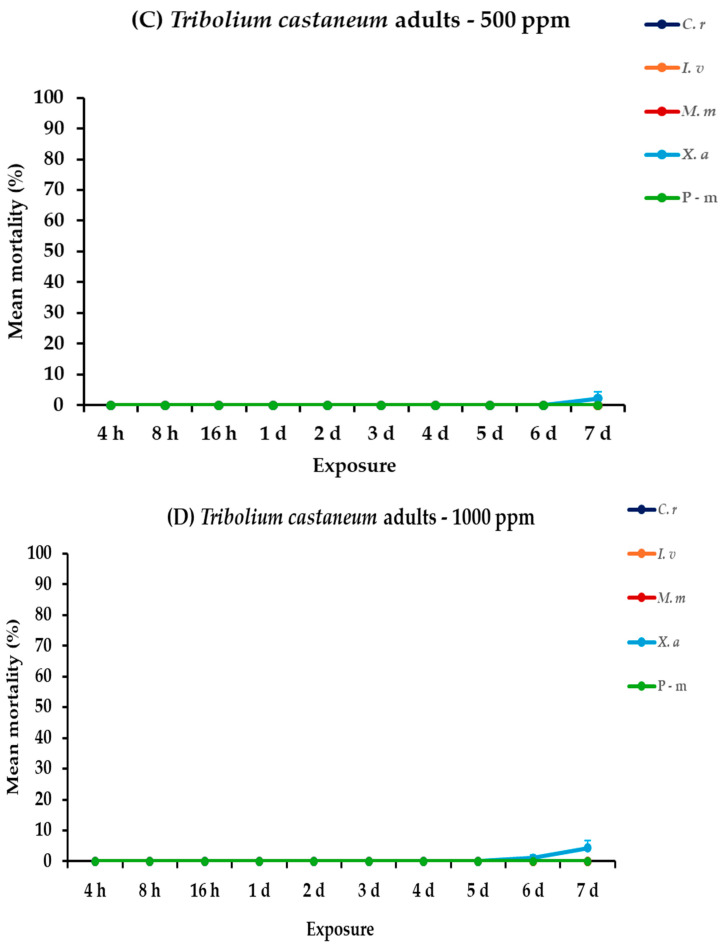
Mean (%) mortality + standard error (SE) of *Tribolium castaneum* larvae (**A**,**B**) and adults (**C**,**D**) after 4–16 h and 1–7 days in wheat treated with *C. reticulata*, *I. verum*, *M. myristica*, and *X. aethiopica* (under the abbreviations *C. r*, *I. v*, *M. m*, and *X. a*, respectively) EOs at two concentrations and pirimiphos-methyl (under the abbreviation P-m) as positive control.

The analysis of mortality rates for *T. castaneum* larvae and adults revealed that all the main effects and their interactions between and within exposures were significant. For adults, only concentration had no significant effect (Table 2). *Illicium verum*, *X. aethiopica*, and *C. reticulata* EOs were the most effective in controlling *T. castaneum* larvae, particularly at the high concentration. At 500 ppm, *I. verum* showed the highest mortality rate for larvae, reaching 86.7% on day 7, followed closely by *X. aethiopica* with 85.6%, while *C. reticulata* and *M. myristica* exhibited moderate effectiveness, peaking at 56.7 and 24.4%, respectively. The positive control, P-m, achieved 58.9% mortality on day 7. At 1000 ppm, complete mortality (100%) was displayed by *I. verum* on day 5, and by *C. reticulata* and *X. aethiopica* on day 7. *Monodora myristica* demonstrated a substantial increase in effectiveness at this concentration, attaining 52.2% mortality on day 7. In contrast, the adult *T. castaneum* showed zero to minimal mortality rates to all treatments at both concentrations, with *X. aethiopica* at 1000 ppm resulting only at 4.4% by the last day of the bioassay (Figure 2, Table S2).

### 2.4. Effectiveness Against T. confusum Adults and Larvae

**Figure 3:**
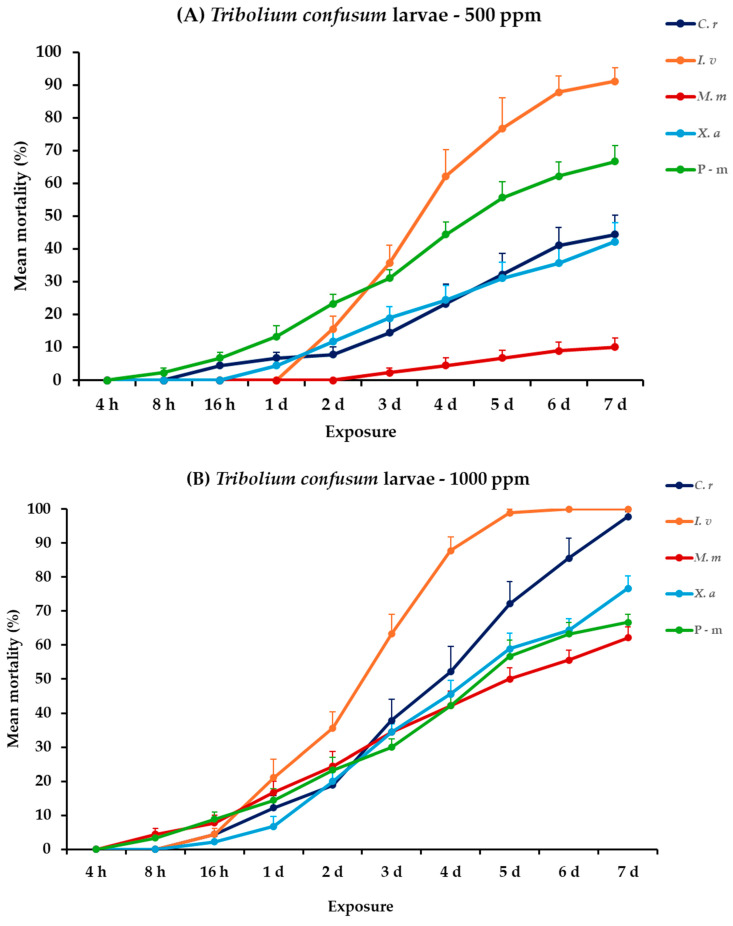

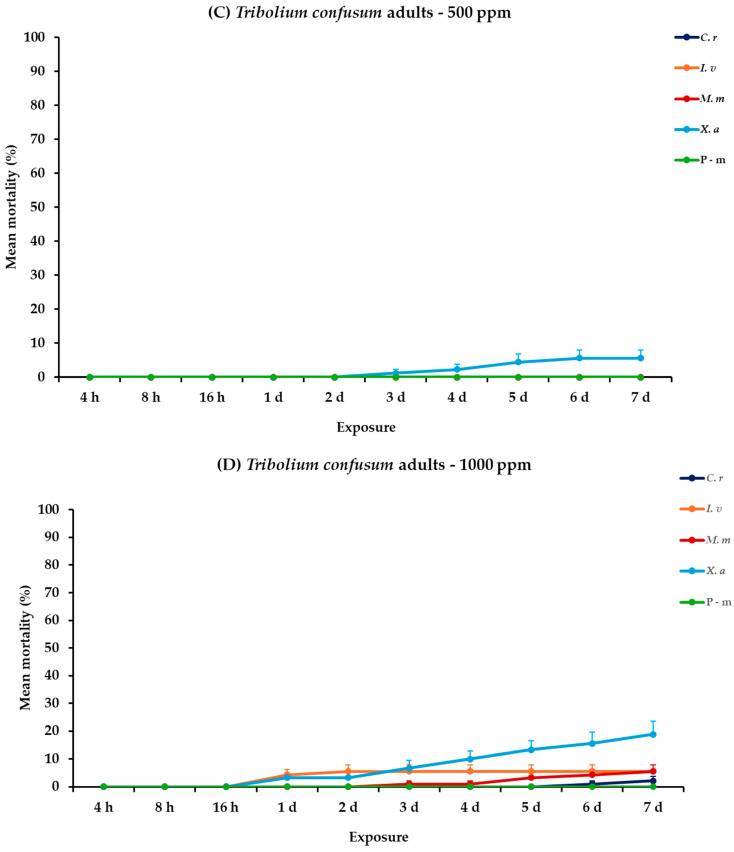
Mean (%) mortality + standard error (SE) of *Tribolium confusum* larvae (**A**,**B**) and adults (**C**,**D**) after 4–16 h and 1–7 days in wheat treated with *C. reticulata*, *I. verum*, *M. myristica*, and *X. aethiopica* (under the abbreviations *C. r*, *I. v*, *M. m*, and *X. a*, respectively) EOs at two concentrations and pirimiphos-methyl (under the abbreviation P-m) as positive control.

For *T. confusum* larvae, all the main effects and respective interactions between and within exposures were significant. For adults, all main effects were significant between exposures, except concentration × EO type. Within exposures the main effects and their interactions were significant (Table 2). *Citrus reticulata* caused an initial larval mortality of 4.4% at 16 h, eventually reaching 44.4% on day 7 at 500 ppm. Nevertheless, *I. verum* demonstrated a rapid and significant increase in mortality, achieving 91.1% on day 7 post-exposure. *Monodora myristica* and *X. aethiopica* were less effective, with 10 and 42.2% mortality on the last day, respectively. At the highest concentration, the effectiveness of the EOs significantly improved. Specifically, on day 6, *I. verum* caused complete mortality (100%). On day 7, *C. reticulata* caused a near-total mortality of 97.8%, while *M. myristica* and *X. aethiopica* showed elevated efficacy with rates of 62.2 and 76.7% mortality, respectively. The positive control caused the same level of mortality (66.7%) either compared to EOs tested at 500 ppm or 1000 ppm. For adults of *T. confusum*, at 500 ppm, only *X. aethiopica* exhibited slight activity, reaching 5.6%, whereas the other EOs, including the positive control, P-m, showed no significant mortality. At 1000 ppm, again *X. aethiopica* displayed a visible increase and peaked at 18.9%; meanwhile, the rest of the EOs showed minimal lethal effects, with recorded mortality remaining below 6% (Figure 3, Table S3).

### 2.5. Effectiveness Against T. molitor Adults and Larvae

**Figure 4:**
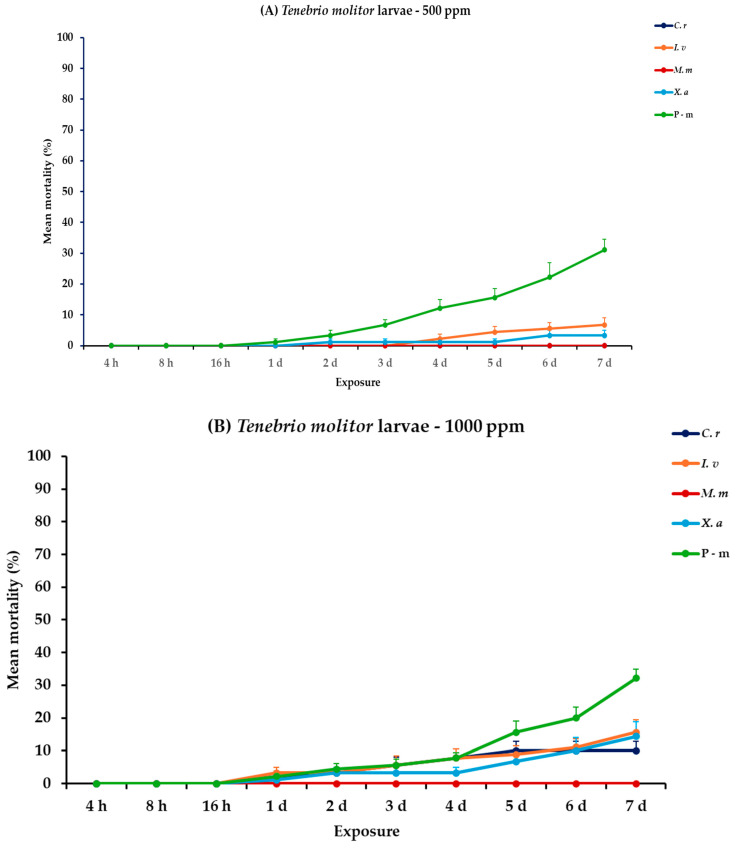

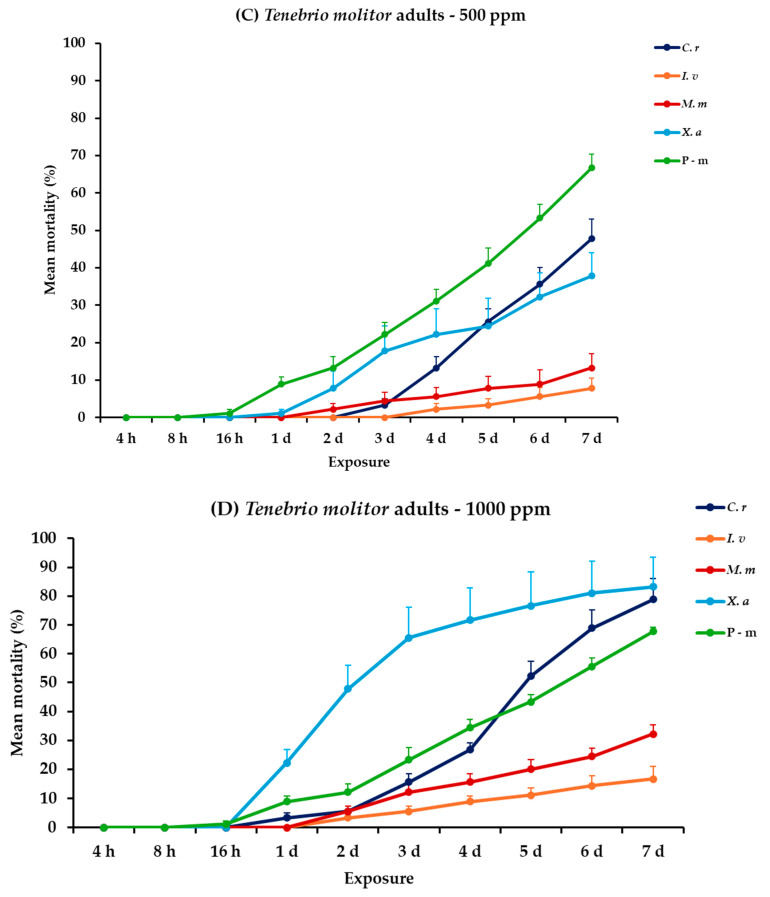
Mean (%) mortality + standard error (SE) of *Tenebrio molitor* larvae (**A**,**B**) and adults (**C**,**D**) after 4–16 h and 1–7 days in wheat treated with *C. reticulata*, *I. verum*, *M. myristica*, and *X. aethiopica* (under the abbreviations *C. r*, *I. v*, *M. m*, and *X. a*, respectively) EOs at two concentrations and pirimiphos-methyl (under the abbreviation P-m) as positive control.

For the analysis of the adults and larvae of *T. molitor*, all main effects and respective interactions were significant both between and within exposures for adults. For larvae, the main effect exposure × concentration and the interaction exposure × concentration × EO type had no significant effect (Table 2). At 500 ppm, larvae exhibited minimal mortality rates across all EO treatments (<6.7%), with the only significant increase being observed with P-m on the last day (31.1%). In contrast, adult mortality was significantly more pronounced, particularly with *C. reticulata* and *X. aethiopica* displaying the highest mortality rate at 47.8 and 37.8%, respectively, on day 7, without surpassing the P-m rate of 66.7%. A similar trend was detected at 1000 ppm, both larvae and adults displayed higher but still moderate mortality values, notably with *C. reticulata* larvae reaching 10.0% and adults peaking at 78.9% on day 7. Similarly, *X. aethiopica* displayed a distinct difference between larvae and adults, with 14.4 and 83.3% on day 7, respectively (Figure 4, Table S4).

### 2.6. Effectiveness Against T. granarium Adults and Larvae

**Figure 5:**
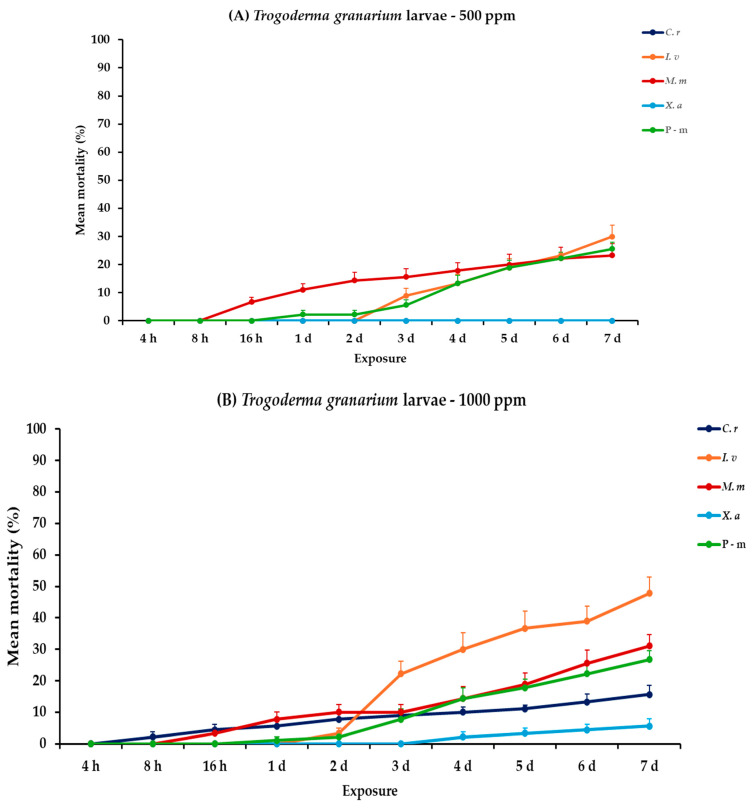

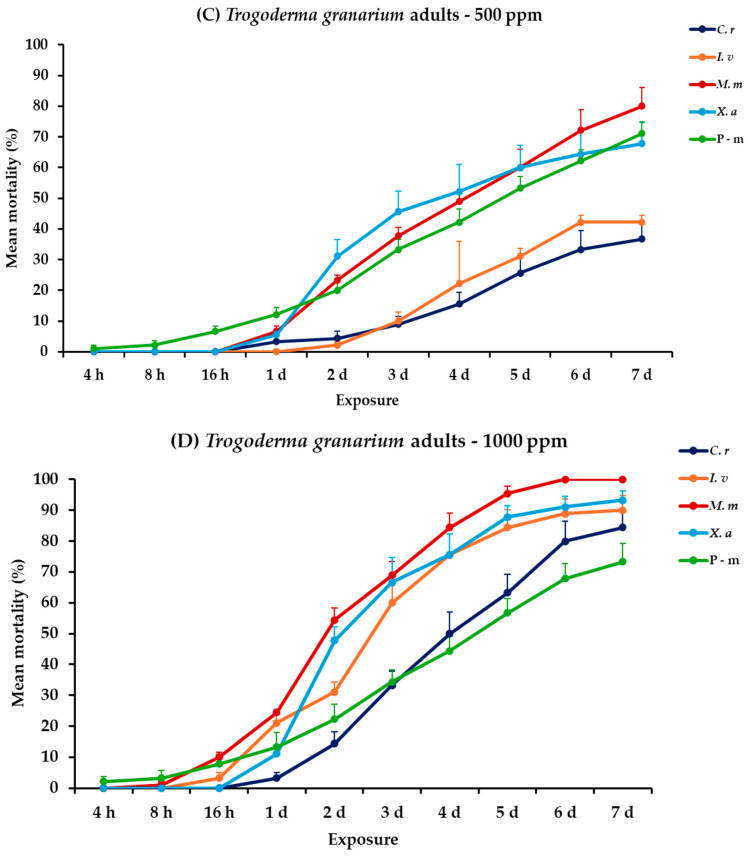
Mean (%) mortality + standard error (SE) of *Trogoderma granarium* larvae (**A**,**B**) and adults (**C**,**D**) after 4–16 h and 1–7 days in wheat treated with *C. reticulata*, *I. verum*, *M. myristica*, and *X. aethiopica* (under the abbreviations *C. r*, *I. v*, *M. m*, and *X. a*, respectively) EOs at two concentrations and pirimiphos-methyl (under the abbreviation P-m) as positive control.

For *T. granarium*, all main effects and corresponding interactions were significant for adults and larvae, both within and between exposures (Table 2). At the lower concentration, larval mortality remained low at 7 days post-exposure. *Citrus reticulata* and *X. aethiopica* showed no significant larval mortality at this concentration. Recorded data for *I. verum* and *M. myristica* were initially minimal and increased consistently to 30.0 and 23.3%, respectively. For adults of *T. granarium*, mortality increased substantially with time. *Monodora myristica* achieved the highest adult mortality of 80.0%, followed by *X. aethiopica* (67.8%) at 7 days post-exposure. At 1000 ppm, a slight increase but no significant difference was observed in larval mortality for both *I. verum* and *M. myristica* (47.8 and 31.1%, respectively), while *X. aethiopica* exhibited minimal efficacy (5.6%). Recorded larval mortality of positive control, when compared to EOs tested at 500 or 1000 ppm, also remained stable at approximately 25.0%. Adult mortality reached 100% for *M. myristica* after 6 days, followed by *X. aethiopica* (93.3%), *I. verum* (90.0%), and *C. reticulata* (84.4%) at the end of the exposure period. The positive control in both exposure trials also yielded relatively lower results but remained high, with approximately 70% mortality (Figure 5, Table S5).

### 2.7. Effectiveness Against O. surinamensis Adults and Larvae

**Figure 6:**
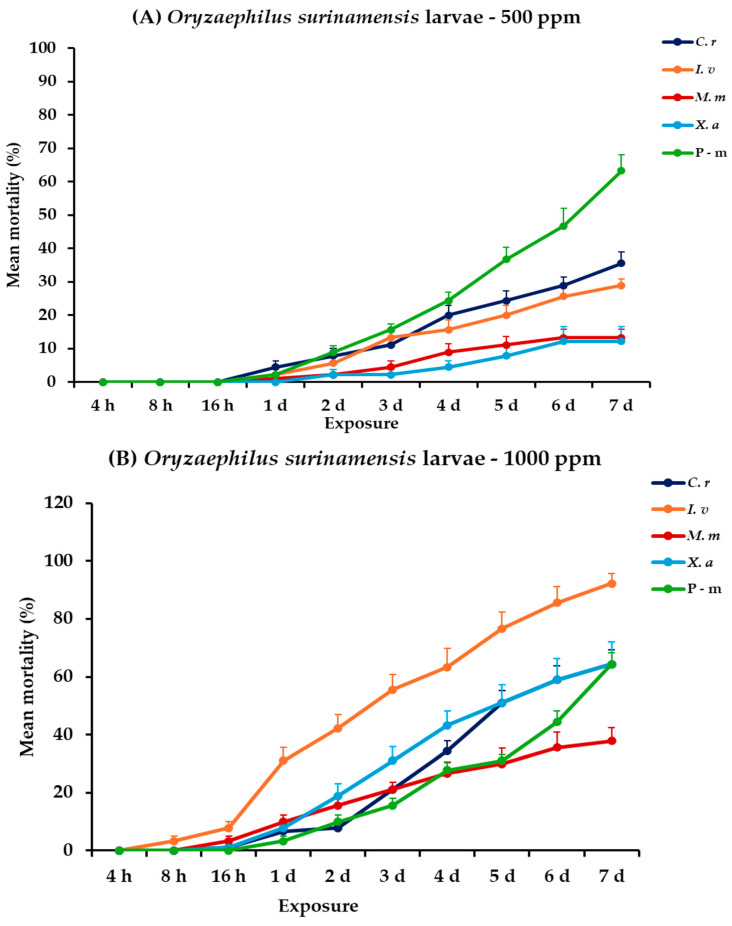

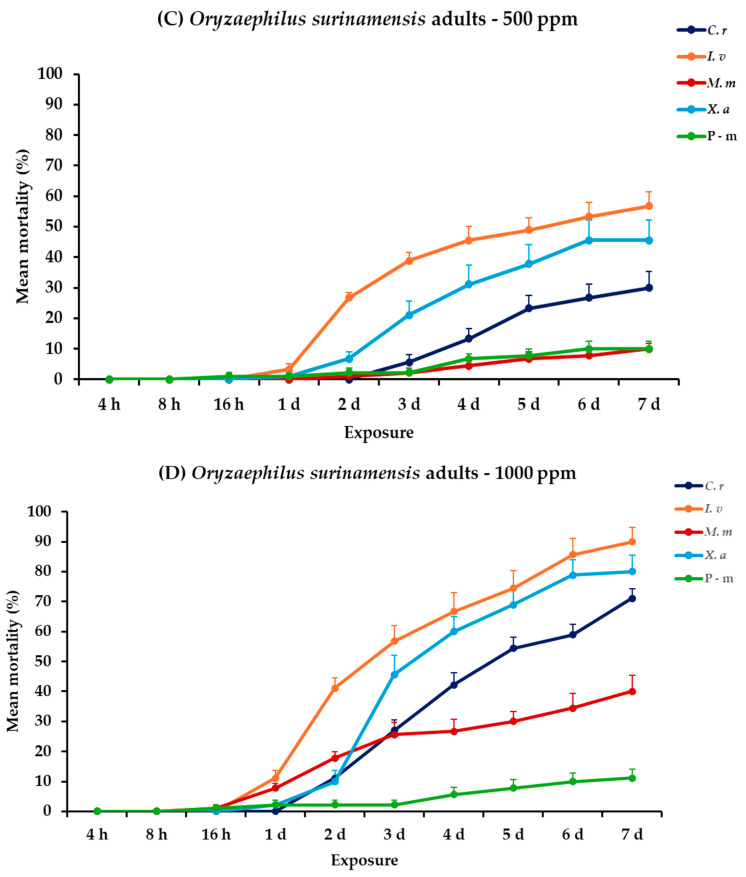
Mean (%) mortality + standard error (SE) of *Oryzaephilus surinamensis* larvae (**A**,**B**) and adults (**C**,**D**) after 4–16 h and 1–7 days in wheat treated with *C. reticulata*, *I. verum*, *M. myristica*, and *X. aethiopica* (under the abbreviations *C. r*, *I. v*, *M. m*, and *X. a*, respectively) EOs at two concentrations and pirimiphos-methyl (under the abbreviation P-m) as positive control.

For *O. surinamensis*, significance was demonstrated for all the main effects and their respective interactions for both life stages, within and between exposures, except the exposure × concentration × EO type interaction for adults (Table 2). At 500 ppm, larval mortality increased gradually over 7 days for all EOs, with *C. reticulata* and *I. verum* demonstrating relatively moderate mortality rates at the end of the experiment (35.6 and 28.9%, respectively), though none approached the 63.3% mortality observed with P-m. Adult mortality trends at 500 ppm suggested that *I. verum* and *X. aethiopica* were the most lethal, achieving 56.7 and 45.6% mortality, respectively, at the last day, whereas P-m remained at 10%. At 1000 ppm, larval mortality was significantly higher. Specifically, *I. verum* caused 92.2%, followed by *X. aethiopica* and *C. reticulata*, which both peaked at 64.4% after a seven-day period. Accordingly, *I. verum* also exhibited the highest mortality for adults of this species, reaching a high percentage (90%), while *C. reticulata* and *X. aethiopica* also showed a significant increase in effectiveness (71.1 and 80%, respectively) (Figure 6, Table S6).

### 2.8. Effectiveness Against R. dominica Adults

**Figure 7:**
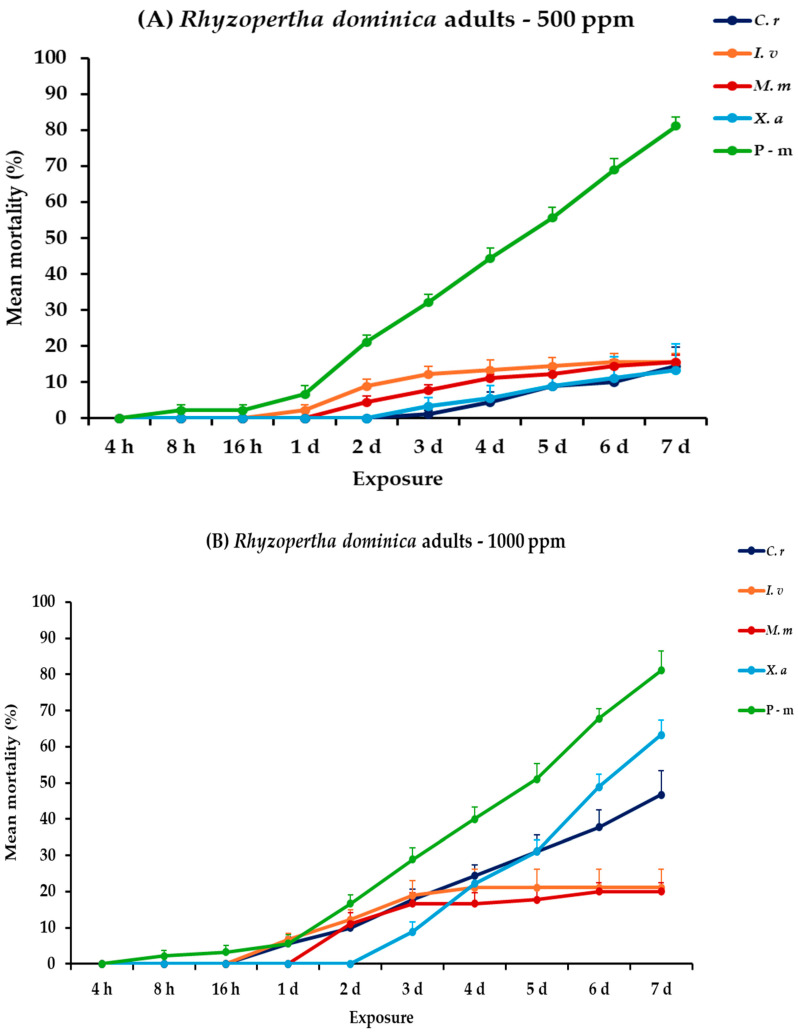
Mean (%) mortality + standard error (SE) of *Rhyzopertha dominica* adults (**A**,**B**) after 4–16 h and 1–7 days in wheat treated with *C. reticulata*, *I. verum*, *M. myristica*, and *X. aethiopica* (under the abbreviations *C. r*, *I. v*, *M. m*, and *X. a*, respectively) EOs at two concentrations and pirimiphos-methyl (under the abbreviation P-m) as positive control.

For *R. dominica* adults, all main effects were significant, along with their associated interactions, between and within exposures (Table 2). At 500 ppm of tested EOs, *C. reticulata* and *X. aethiopica* exhibited minimal efficacy, with mortality rates remaining below 15% even after the 7-day period. Additionally, *I. verum* and *M. myristica* showed slightly higher mortality rates, both reaching up to 15.6% on the seventh day. At 1000 ppm, all EOs procured elevated mortality percentages in correlation with exposure time. *Citrus reticulata* and *X. aethiopica* exhibited substantial increases in mortality, up to 46.7 and 63.3%, respectively, on day 7, while *I. verum* and *M. myristica* showed low improvements in mortality, reaching 21.1 and 20.0%, respectively, without significant differences. Interestingly, P-m remained the most effective treatment against this species, achieving 81.1% mortality rate either compared to EOs tested at 500 or 1000 ppm (Figure 7, Table S7).

### 2.9. Effectiveness Against S. oryzae Adults

**Figure 8:**
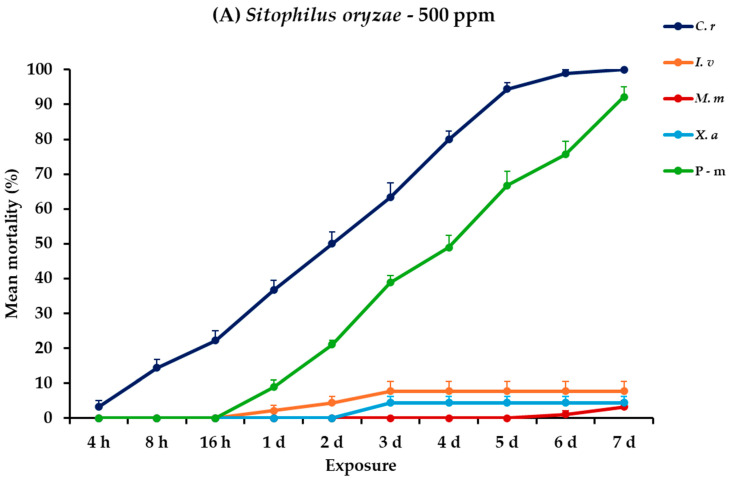

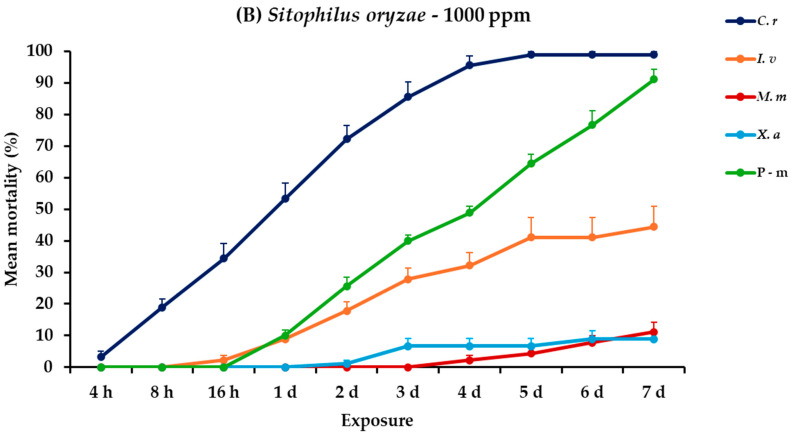
Mean (%) mortality + standard error (SE) of *Sitophilus oryzae* adults (**A**,**B**) after 4–16 h and 1–7 days in wheat treated with *C. reticulata*, *I. verum*, *M. myristica*, and *X. aethiopica* (under the abbreviations *C. r*, *I. v*, *M. m*, and *X. a*, respectively) EOs at two concentrations and pirimiphos-methyl (under the abbreviation P-m) as positive control.

For *S. oryzae* adults, all main effects and their interactions across different exposure times were significant (Table 2). At 500 ppm, *C. reticulata* showed a marked increase in mortality, reaching 100% at the end of the trial. Contrarily, *I. verum*, *M. myristica*, and *X. aethiopica* exhibited significantly lower effectiveness, with mortalities peaking at 7.8, 3.3, and 4.4%, respectively. At the higher concentration, the efficacy of *C. reticulata* remained high, achieving 98.9% mortality. Following *C. reticulata*, *I. verum* showed improved results at this concentration, with mortality rates rising to 44.4% on day 7, whereas *M. myristica* and *X. aethiopica* continued to show limited effectiveness, with recorded mortalities of 11.1 and 8.9%, respectively, on day 7. Positive control also remained highly effective at both dose scenarios against *S oryzae*, achieving mortality rates of approximately 90% (Figure 8, Table S8).

### 2.10. Effectiveness Against A. siro Adults and Nymphs

**Figure 9:**
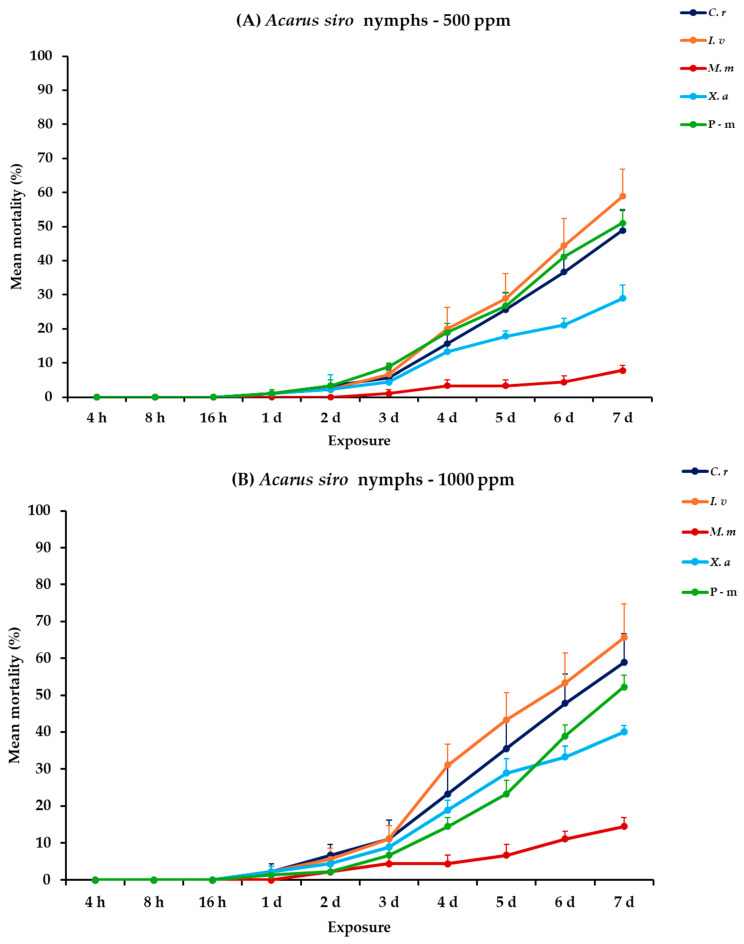

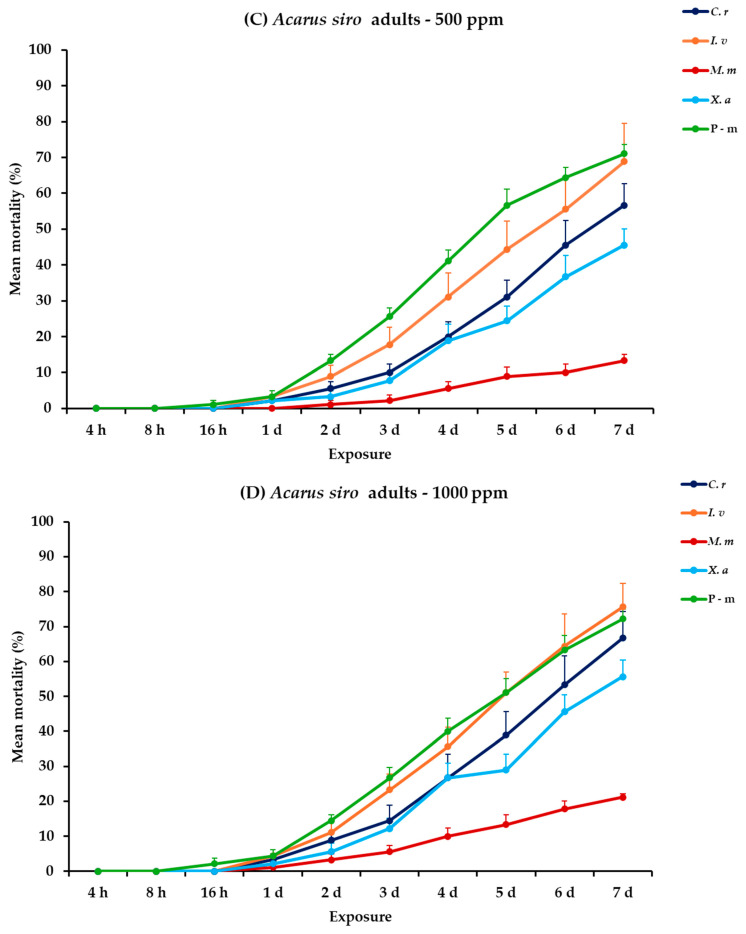
Mean (%) mortality + standard error (SE) of *Acarus siro* nymphs (**A**,**B**) and adults (**C**,**D**) after 4–16 h and 1–7 days in wheat treated with *C. reticulata*, *I. verum*, *M. myristica*, and *X. aethiopica* (under the abbreviations *C. r*, *I. v*, *M. m*, and *X. a*, respectively) EOs at two concentrations and pirimiphos-methyl (under the abbreviation P-m) as positive control.

For *A. siro* nymphs and adults, all main effects exhibited significance between exposures, apart from the concentration × EO type interaction. Within exposures, significance was displayed for only the exposure and exposure × EO type interaction (Table 2). For nymphs at 500 ppm, *C. reticulata* and *I. verum* demonstrated increasing mortality from initially 1.1% on day 1 to 48.9 and 58.9% on day 7, respectively. On the contrary, *M. monodora* and *X. aethiopica* showed significantly lower mortality rates, reaching 7.8 and 28.9%, respectively. Furthermore, at 1000 ppm, *C. reticulata* and *I. verum* maintained similar efficacy with the previous concentration with moderate results of 58.9 and 65.6%, respectively. Moreover, *M. monodora* and *X. aethiopica* demonstrated a slight elevation in efficacy at the end of the experiment (14.4 and 40.0%, respectively). Concerning *A. siro* adults, *C. reticulata*, *I. verum* and *X. aethiopica* caused significantly higher mortalities than *M. monodora*, reaching 68.9% after 7 d at 500 ppm. At the highest concentration (1000 ppm), mortality rates increased for all tested EOs, with *I. verum* killing 75.6%, followed by *C. reticulata* (66.7%), *X. aethiopica* (55.6%), and *M. monodora* (21.1%), at 7 d. Lastly, P-m caused approximately 50.0 and 70.0% nymphal and adult mortality, respectively, on day 7 either compared to EOs tested at 500 or 1000 ppm (Figure 9, Table S9).

## 3. Discussion

The EOs employed in this study were chemically characterized by GC-MS. More details on the chemical composition of *M. myristica* and *X. aethiopica* can be found in the work of Wandjou et al. [45]. Regarding *I. verum*, the chemical composition of its EO was quite linear with those previously published. Indeed, this EO is commonly characterized by phenylpropanoids, mainly represented by (*E*)-anethole, with average percentages ranging from 79.9 to 92.4% [49,50,51,52,53]. On the other hand, the chemical composition of *C. reticulata* EO, which was dominated by limonene, is consistent with those already published. Indeed, it is commonly characterized by high percentages of this compound, ranging from 76 to 85% [54,55]. However, the chemical composition of the EO varies according to factors such as plant part used, extraction technique, developmental stage, and environmental conditions [56].

The present study highlights the pesticidal effects of EOs derived from *I. verum*, *C. reticulata*, *M. myristica*, and *X. aethiopica* against several key arthropod pests of stored products. Among the four botanical EOs tested, those from *I. verum* and *C. reticulata* demonstrated the highest toxic activity, particularly against larval stages, where complete mortality was often achieved. Notably, *I. verum* EO was perticularly effective at the highest concentration, showing pronounced mortality across most pest species, although *T. molitor* larvae exhibited a negligible response. Similarly, *C. reticulata* EO displayed substantial efficacy, achieving total mortality against *A. diaperinus*, *T. castaneum* larvae, and *S. oryzae* adults. These findings align with previous reports of contact, fumigation, and repellency bioassays, where *I. verum* demonstrated strong activity against important insect species such as *Sitophilus zeamais* (Motschulsky) (Coleoptera: Curculionidae), *Cryptolestes ferrugineus* (Stephens) (Coleoptera: Laemophloeidae), and *A. diaperinus* [57,58,59,60]. A clear trend emerged from our study, indicating that higher concentrations and longer exposure times were directly correlated with increased mortality. This time- and concentration-dependent effect is consistent with other studies, such as that of Matos et al. [61], who reported that *I. verum* exhibited strong fumigant efficacy against *Callosobruchus maculatus* (F.) (Coleoptera: Chrysomelidae) at a relatively low lethal concentration (LC_50_ = 22.36 μL/L). Furthermore, in contact and repellency assays in the same study, *I. verum* was shown to reduce oviposition and inhibit insect emergence, highlighting its multifunctional potential. Time-dependent effects were also demonstrated from Ho et al. [43], reporting a notably higher mortality rate of *T castaneum* at 21 days exposure (69.5%) to *I. verum*-treated rice vs. 7 days (0.5%).

Citrus-derived EOs have also been studied for their insecticidal activity against significant pest species. Fouad and da Camara [62] documented that *C. reticulata* peel EO exhibited significantly higher insecticidal activity against *S. zeamais* adults across multiple bioassays, including contact, ingestion, and fumigant trials, when compared to *Citrus aurantiifolia* (Christm.) Swingle (Sapindales: Rutaceae) EO, with more pronounced effects observed after just one day of exposure. In a fumigant assay conducted by Safavi and Mobki [63], *C. reticulata* EO, at the highest concentration (63 µL/L air), caused significant insect mortality (79%) after 48 h against *T. castaneum*. Both studies highlighted the improvement in efficacy with increased exposure time and dose, further supporting the trend observed in our study. The promising pesticidal action of *C. reticulata* is linked to the presence of limonene. The latter has been reported for its potent anti-acetylcholinesterase (AChE) activity [64] as well as for its contact toxicity on different pests [65,66,67,68,69]. Regarding *I. verum* EO, its pesticidal action is due to the high content of (*E*)-anethole. This phenylpropanoid has been reported for the inhibition of AChE activity [59], thus affecting the normal neurological activity of the insect. Additionally, it is well known that phenylpropanoids affect the insect defense system neutralizing enzymes such as P450, esterases, or glutathione-S-transferases) [70].

Despite the overall effectiveness of the tested EOs, *M. myristica* and *X. aethiopica* exhibited comparatively lower efficacy, with mortality rates generally below 50%. However, *M. myristica* achieved complete mortality in *T. granarium* adults at 1000 ppm on day 6, while *X. aethiopica* was lethal to the larvae of both *Tribolium* species and *T. molitor* adults. Similarly, × *Hesperotropsis leylandii* (A.B. Jacks. and Dallim.) Garland and G. Moore (Cupressales: Cupressaceae) E.O. was 100% lethal to the larvae of both *T. confusum* and *T. molitor* species at 1000 ppm exposure by day 7, while adults remained completely unaffected. Likewise, *Juniperus* × *pfitzeriana* (Spath) P.A. Schmidt (Cupressales: Cupressaceae) achieved 100% mortality in *T. granarium* adults at 1000 ppm by day 7, while less than 6% in larvae [12]. However, after the exposure to treated millet seeds with a notably higher concentration of *X. aethiopica* (40,000 ppm), *T. castaneum* adults achieved a greater mortality rate (68.3%) [44], compared to the present study (4.4% at 1000 ppm). These findings provide valuable data that can help develop targeted applications for different pest species and their developmental stages.

The different susceptibility observed between larvae and adults in our study emphasizes the importance of targeting specific life stages in pest management. Although larvae were generally more susceptible to higher EO concentrations, previous research has indicated that *T. molitor* adults can sometimes be more vulnerable to botanical treatments than larvae [11,71]. This discrepancy may stem from species- or treatment-specific differences, reflecting the complexity of insect physiology in response to different compounds. Physiological factors contributing to this response could include variations in cuticle structure between species [72,73].

EOs have gained recognition as effective botanical active ingredients in pest control across several agricultural and urban settings [74,75,76]. Their bioactivity, low toxicity to non-target organisms, high volatility, and sensitivity to temperature and UV light degradation make them eco-friendly alternatives to synthetic insecticides [77,78]. The active components in EOs are recognized for their pesticidal, antioxidant, and antimicrobial properties, contributing to their effectiveness in biological applications [79]. While synthetic insecticides, such as organophosphates and pyrethroids, have proven effective in managing stored-product pests [80,81], reliance on them raises concerns due to resistance development and environmental risks from chemical residues [82]. Many pests, such as *Tribolium* spp., *S. oryzae*, *A. diaperinus*, and *R. dominica*, have developed resistance to these chemicals [83,84,85,86,87,88,89,90,91]. Even mite pests like *A. siro* have shown resistance to organophosphates, such as pirimiphos-methyl, which has been used for decades as a grain protectant [92,93]. Interestingly, natural pesticides like *Carlina acaulis* L. (Asterales: Asteraceae) EO have demonstrated significant efficacy against both life stages of *A. siro* in contact bioassays, achieving approximately 90% mortality [94]. In our study, *A. siro* adults were more susceptible to 1000 ppm of *I. verum* and *X. aethiopica* (>70% mortality) EO than to the positive control (~50% mortality), suggesting that they can be effective against both insect and mite species occurring in stored-product facilities.

Research on *Tribolium* species has focused on overcoming the challenge of controlling these pests due to the increasing incidence of resistant strains worldwide [95,96,97]. One of the notable findings of our study is the susceptibility of larval individuals of both *Tribolium* species to three EOs, which could prove essential for developing more environmentally sustainable pest management strategies. In this study, pirimiphos-methyl, used as a positive control, peaked at 66.7% mortality, often remaining below this rate. Notably, zero toxicity was recorded against the adult stages of the two *Tribolium* species, further highlighting the declining efficacy of conventional insecticides.

The outcomes of this research emphasize the significant potential of EOs, particularly those from *I. verum* and *C. reticulata*, in surpassing the performance of the conventional synthetic insecticide pirimiphos-methyl. These findings advocate for the inclusion of specific EOs in integrated pest management (IPM) approaches, providing a more sustainable and eco-friendlier alternative with reduced environmental consequences. Future research should focus on the development and optimization of EO-based formulations, ensuring their long-term efficacy under different storage conditions. This type of research should also focus on the commercialization of EOs as effective insecticides for the management of stored-product pests and potential replacement of chemicals, chiefly organophosphates and pyrethroids. Our study clearly showed that in several cases the tested EOs outperformed pirimiphos-methyl, during the followed short exposure scenario, against *A. diaperinus* larvae, *T. castaneum* larvae, *Tribolium confusum* larvae, *Tenebrio molitor* adults, *Trogoderma granarium* larvae and adults, *O. surinamensis* larvae or adults, *S. oryzae* adults, and *A. siro* nymphs or adults. This knowledge should be considered when research involves steps for commercial development of EOs as insecticides in storages. Investigating potential synergies with other biological or chemical controls to counteract resistance development will also be crucial. Finally, expanding these studies to cover a broader spectrum of pests and conducting environmental assessments will solidify the role of EOs in managing resistant pest populations in stored-product systems.

## 4. Materials and Methods

### 4.1. Essential Oils (EOs) and Their Chemical Analysis in GC-MS

*Illicium verum* EO was kindly provided by FD Copeland & Sons Limited (batch P61T, 10/10/07) (London, UK), while *X. aethiopica* and *M. myristica* EOs were obtained as previously described in the work of Wandjou et al. [45]. As regards *C. reticulata* EO, it was obtained from *C. reticulata* peels deriving from commercial fruits. In detail, dry peels (1100 g) were placed in a 20 L round flask with 11 L of distilled water and subjected to hydrodistillation using a Clevenger-type apparatus for 4 h. The system was heated using a mantle system Falc MA (Falc Instruments, Treviglio, Italy). The EO presented a pale-yellow color and was obtained with a yield of 2.9% (*w*/*w*). *Xylopia aethiopica* and *M. myristica* EOs were obtained as previously described in the work of Wandjou et al. [45].

The EOs chemical composition was determined by gas chromatography-mass spectrometry analysis (GC-MS) employing the analytical method previously reported by Wandjou et al. [45]. Specifically, the instrument was an Agilent 6890N equipped with a 5973N MS system working at 70 eV in the EI mode (Agilent Technologies, Santa Clara, California, USA). The separation of compounds was achieved through an HP-5MS capillary column (5% phenylmethylpolysiloxane, 30 m length, 0.25 mm internal diameter, and 0.1 µm film thickness). The identification of compounds was achieved as previously published [45].

### 4.2. Commodity

For the bioassays, pesticide-free and uninfested hard wheat, *Triticum durum* Desf. (Poales: Poales) var. Claudio, was implemented. The grain moisture content of the wheat was measured at 12.2% using a moisture meter (mini-GAC plus, Dickey-John Europe S.A.S., Colombes, France) immediately before the assays.

### 4.3. Arthropod Species and Rearing Media

The arthropod individuals utilized in this study were collected from colonies maintained at the Laboratory of Agricultural Zoology and Entomology (LAZE), Agricultural University of Athens, Greece. The colonies were reared under controlled conditions in total darkness. *Acarus siro* was reared at 25 °C with 80% relative humidity (RH), while all other insect species were maintained at 30 °C with 65% RH. The rearing medium for *A. siro* constituted a blend of wheat, flaked oats, and brewer’s yeast in a 10:10:1 ratio. *Rhyzopertha dominica* and *S. oryzae* were reared on whole wheat grains. *Tribolium* spp. were reared in wheat flour with 5% brewer’s yeast. *Tenebrio molitor* was kept in a medium of oat bran and potato cubes to maintain moisture, while *A. diaperinus* was reared on wheat bran with brewer’s yeast and sliced apples for moisture. Finally, *O. surinamensis* was reared on a mixture of processed wheat, oat flakes, and yeast in a ratio of 5:5:1.

### 4.4. Experimental Procedure and Arthropod Selection

Adults of *T. molitor*, both *Tribolium* species, *S. oryzae*, *O. surinamensis*, and *R. dominica* were all under 14 days old, while *T. granarium* adults were <24 h old. *Alphitobius diaperinus* adults were younger than one week. The larvae of *O. surinamensis* and *Tribolium* spp. were used during their 3rd to 4th instar stage, with *T. molitor* larvae measuring 1 to 1.4 cm in length, *T. granarium* larvae ranging between 2–4 mm, and *A. diaperinus* larvae less than 0.7 cm in length. *Acarus siro* individuals, whose sex was not determined, were from colonies between 1 and 21 days old. Nymphs and adults of *A. siro* were distinguished based on external characteristics, specifically the shorter body setae found in nymphs [12].

The selection of the two concentrations tested (500 and 1000 μL EO/kg wheat) was reached after the conduction of preliminary screening trials. Solutions were prepared, and the wheat grains were treated following the methods described in Kavallieratos et al. [94]. Additional batches of wheat were treated separately with pure ethanol, water, or ethanol mixed with Tween 80 plus water as negative controls. Pirimiphos-methyl (Actellic EC 50%) at the commercially recommended dose served as the positive control (5 μL/kg grain). From each treated lot (controls or EOs), 1 g for mites or 10 g for insects were sampled using different scoops. Each wheat sample was placed individually on a Precisa XB3200D laboratory scale (Alpha Analytical Instruments, Gerakas, Greece) for weighing and transported inside glass vials (6.0 × 2.7 cm for *A. siro* and 12.5 × 7.5 cm for the insects). Small vials were sealed with perforated cover lids to ensure adequate aeration, while larger vials were equipped with lids equipped with a 1.5 cm circular opening covered with gauze [98].

Ten arthropods per species and life stage were located in separate vials. To prevent escape, a polytetrafluoroethylene coating was set on the inner portion of the vials close to the lids (Sigma-Aldrich Chemie GmbH, Schnelldorf, Germany). All vials were incubated at 25 °C/80% RH for *A. siro* and 30 °C/65% relative humidity for insect species until the tests were completed. Due to the cannibalistic behavior of *A. diaperinus* [12], 10 vials containing one individual each were used, constituting one sub-replication. Mortality assessments were conducted under a stereoscope, checking for movement at 4, 8, and 16 h, and from 1 to 7 days. Arthropods were gently prodded with a small fine brush to check for any sign of movement, using separate brushes for each type of essential oil and positive or negative controls. Three replications, each with three sub-replications for each arthropod, were performed, with new solutions, wheat samples, and arthropods prepared each time [13].

### 4.5. Data Analysis

No adjustments were required for the control mortality, as it remained below 5% for all tested species. To normalize variance the dataset was transformed by implementing the formula log (x + 1) beforehand [99]. Data analysis for each pest species or developmental stage was conducted using a repeated measures model [100], incorporating interactions of main effects. Exposure, mortality, and EO type/concentrations were the response variable, the repeated factor, and the main effects, respectively. All analyses were performed using JMP 16.2 [101]. Mean separation was conducted using the Tukey HSD test at a significance level of 0.05 [102].

## Figures and Tables

**Table 1:** Chemical composition of the essential oils (EOs) determined by GC-MS analysis.

Component ^b^	RI ^c^	RI Lit. ^d^	*Monodora myristica* ^e^	*Xylopia aethiopica* ^e^	*Ilicium verum*	*Citrus reticulata*	ID ^f^
				Area % ± SD ^a^			
*α*-thujene	920	924	3.1 ± 0.6	1.9 ± 0.4	-	-	RI ^f^, MS ^f^
*α*-pinene	925	932	7.6 ± 1.2	9.6 ± 1.6	0.6 ± 0.17	0.5 ± 0.0	Std
camphene	938	946	-	0.1 ± 0.0	-	-	Std
thuja-2,4(10)-diene	944	953	-	tr ^g^	-	-	RI, MS
sabinene	966	969	0.1 ± 0.0	26.1 ± 3.1	-	-	Std
*β*-pinene	968	974	0.3 ± 0.0	17.4 ± 1.9	-	0.2 ± 0.0	Std
myrcene	988	988	4.3 ± 0.8	0.2 ± 0.0	-	1.0 ± 0.0	Std
*δ*-2-carene	998	1001	0.9 ± 0.0	-	-	-	RI, MS
*α*-phellandrene	1002	1002	32.3 ± 3.6	0.3 ± 0.0	0.2 ± 0.0	-	Std
*δ*-3-carene	1004	1008	-	-	0.2 ± 0.0	-	RI, MS
*α*-terpinene	1013	1014	0.1 ± 0.0	1.9 ± 0.4	-	-	Std
*p*-cymene	1021	1020	32.8 ± 3.0	1.1 ± 0.2	0.1 ± 0.0	-	Std
sylvestrene	1022	1025	-	-	1.7 ± 0.1	-	RI, MS
limonene	1024	1024	4.4 ± 0.9	-	-	97.1 ± 0.1	Std
*β*-phellandrene	1024	1025	-	6.2 ± 1.2	-	-	Std
1,8-cineole	1026	1026	-	3.6 ± 0.7	0.2 ± 0.0	-	Std
(*Z*)-*β*-ocimene	1036	1032	0.3 ± 0.0	1.2 ± 0.3	-	-	Std
(*E*)-*β*-ocimene	1046	1044	0.1 ± 0.0	0.1 ± 0.0	-	-	Std
*α*-terpinene	1054	1054	0.1 ± 0.0	3.2 ± 0.6	-	-	Std
(*Z*)-sabinene hydrate	1062	1065	-	1.3 ± 0.3	-	-	RI, MS
(Z)-linalool oxide	1069	1067	-	-	-	-	RI, MS
terpinolene	1084	1086	tr	0.6 ± 0.2	-	-	Std
(*E*)-sabinene hydrate	1093	1098	-	0.8 ± 0.2	-	-	RI, MS
linalool	1100	1095	1.9 ± 0.4	0.1 ± 0.0	0.3 ± 0.0	0.2 ± 0.0	Std
(Z)-*p*-menth-2-en-1-ol	1117	1118	0.2 ± 0.0	0.3 ± 0.0	-	-	RI, MS
*α*-campholenal	1122	1122	tr	0.1 ± 0.0	-	-	RI, MS
*allo*-ocimene	1128	1128	-	0.1 ± 0.0	-	-	RI, MS
(*E*)-pinocarveol	1131	1135	-	0.4 ± 0.1	-	-	Std
(*E*)-*p*-menth-2-en-1-ol	1135	1136	0.1 ± 0.0	0.2 ± 0.0	-	-	RI, MS
(*E*)-verbenol	1140	1140	-	0.1 ± 0.0	-	-	RI, MS
pinocarvone	1156	1160	-	0.2 ± 0.0	-	-	RI, MS
borneol	1159	1165	0.1 ± 0.0	tr	-	-	Std
*p*-mentha-1,5-dien-8-ol	1164	1166	-	tr	-	-	RI, MS
(*Z*)-pinocamphone	1167	1172	-	tr	-	-	RI, MS
terpinen-4-ol	1172	1174	-	6.1 ± 1.1	-	0.2 ± 0.1	Std
*p*-cymen-8-ol	1183	1179	0.1 ± 0.0	-	-	-	RI, MS
cryptone	1181	1183	-	0.1 ± 0.0	-	-	RI, MS
*α*-terpineol	1186	1186	0.5 ± 0.1	1.0 ± 0.2	-	0.1 ± 0.0	RI, MS
myrtenal	1189	1195	-	0.2 ± 0.0	-	-	Std
myrtenol	1191	1194	-	0.4 ± 0.1	-	-	Std
methyl chavicol	1192	1195	-	-	3.2 ± 0.0	-	RI, MS
(*E*)-piperitol	1203	1207	0.1 ± 0.0	-	-	-	RI, MS
verbenone	1204	1204	-	0.1 ± 0.0	-	-	RI, MS
cuminaldehyde	1236	1238	-	tr	-	-	RI, MS
neral	1239	1235	-	-	-	-	RI, MS
carvotanacetone	1243	1244	0.1 ± 0.0	-	-	-	RI, MS
*p*-anisaldehyde	1246	1247	-	-	0.5 ± 0.0	-	RI, MS
geraniol	1264	1249	-	-	-	-	Std
geranial	1272	1264	-	-	-	-	Std
(*E*)-anethole	1281	1282	-	-	92.6 ± 0.1	-	Std
carvacrol	1303	1298	0.6 ± 0.2	-	-	-	Std
*δ*-elemene	1331	1335	-	1.8 ± 0.4	-	-	RI, MS
*α*-cubebene	1343	1345	-	0.1 ± 0.0	-	-	RI, MS
*α*-ylangene	1362	1373	-	0.2 ± 0.0	-	-	RI, MS
*α*-copaene	1367	1374	0.1 ± 0.0	0.4 ± 0.1	-	-	RI, MS
*β*-cubebene	1382	1387	-	0.1 ± 0.0	-	-	RI, MS
*β*-elemene	1385	1389	-	0.2 ± 0.0	-	-	Std
geranyl acetate	1385	1379	-	-	-	-	RI, MS
cyperene	1387	1398	-	-	-	-	RI, MS
(*E*)-caryophyllene	1408	1417	-	-	tr	-	Std
(*Z*)-*α*-bergamotene	1408	1411	0.1 ± 0.0	-	-	-	RI, MS
*β*-ylangene	1408	1419	-	0.2 ± 0.0	-	-	RI, MS
*α*-santalene	1412	1416	0.5 ± 0.1	-	-	-	RI, MS
*β*-copaene	1419	1430	-	0.1 ± 0.0	-	-	RI, MS
*γ*-elemene	1426	1434	-	0.3 ± 0.0	-	-	RI, MS
(*E*)-*α*-bergamotene	1430	1432	tr	-	0.1 ± 0.0	-	RI, MS
6,9-guaiadiene	1434	1442	-	0.1 ± 0.0	-	-	RI, MS
*α*-humulene	1442	1452	tr	0.1 ± 0.0	-	-	Std
germacrene D	1471	1484	-	9.7 ± 1.6	-	-	RI, MS
bicyclogermacrene	1486	1500	-	0.5 ± 0.2	-	-	RI, MS
*α*-muurolene	1492	1500	0.1 ± 0.0	tr	-	-	RI, MS
*δ*-amorphene	1498	1511	-	0.1 ± 0.0	-	-	RI, MS
*γ*-cadinene	1504	1513	0.3 ± 0.0	0.1 ± 0.0	-	-	RI, MS
*δ*-cadinene	1516	1522	1.2 ± 0.3	0.3 ± 0.1	-	-	RI, MS
*α*-cadinene	1528	1537	tr	-	-	-	RI, MS
germacrene B	1544	1559	-	0.3 ± 0.1	-	-	RI, MS
*epi*-*α*-cadinol	1631	1638	0.1 ± 0.0	-	-	-	RI, MS
*epi*-*α*-muurolol	1645	1640	0.1 ± 0.0	-	-	-	RI, MS
manool oxide	1991	1987	-	0.1 ± 0.0	-	-	RI, MS
Total identified (%)			92.9	99.6	99.7	99.3	
Monoterpene hydrocarbons			86.5	69.8	2.7	98.8	
Oxygenated monoterpenes			3.8	15.0	0.5	-	
Sesquiterpene hydrocarbons			2.4	14.6	0.1	-	
Oxygenated sesquiterpenes			0.2	-	-	0.5	
Phenylpropanoids			-	-	95.8	-	
Others			tr	0.2	0.5	-	

^a^ Components are ordered according to their elution from the HP-5MS column. ^b^ Linear retention index calculated according to the Van den Dool and Kratz formula. ^c^ Retention index taken from Adams [46]. ^d^ Relative percentage values are mean of two independent analyses: SD, standard deviation. ^e^ These essential oils (EOs) have already been chemically characterized and the composition reported herein is that previously published. ^f^ Identification methods: Std, comparison with available analytical standard; RI, coherence of the calculated RI with those stored in the Adams [46] and NIST 17 [47] libraries; mass spectrum (MS) matching with respect to Adams [46], FFNSC [48], and NIST 17 [47] libraries. ^g^ tr, % < 0.1.

**Table 2:** MANOVA parameters depicting the main effects and their interactions leading to the observed mortalities of *A. diaperinus*, *T. castaneum*, *T. confusum*, *T. molitor*, *T. granarium*, *O. surinamensis* larvae and adults, *R. dominica* and *S. oryzae* adults, and *A. siro* nymphs and adults, between and within exposure intervals (error df = 80 for all pest species and developmental stages).

		Between Exposures				Within Exposures			
Pest Species		Intercept	Concentration	EO Type	Concentration × EO Type	Exposure	Exposure × Concentration	Exposure × EO Type	Exposure × Concentration × EO Type
	df	1	1	4	4	9	9	36	36
*A. diaperinus* larvae	*F*	2715.4	73.8	416.2	11.1	372.9	7.8	27.4	1.6
	*p*	<0.01	<0.01	<0.01	<0.01	<0.01	<0.01	<0.01	0.02
*A. diaperinus* adults	*F*	81.4	0.9	74.5	9.8	128.6	22.4	4.2	0.7
	*p*	<0.01	0.34	<0.01	<0.01	<0.01	<0.01	0.01	0.94
*T. castaneum* larvae	*F*	5407.0	55.1	25.3	11.2	7132.1	21.7	8.7	3.3
	*p*	<0.01	<0.01	<0.01	<0.01	<0.01	<0.01	<0.01	<0.01
*T. castaneum* adults	*F*	4.5	1.3	87.3	27.3	175.4	162.7	20.2	20.1
	*p*	0.04	0.25	<0.01	<0.01	<0.01	<0.01	<0.01	<0.01
*T. confusum* larvae	*F*	2671.7	82.7	47.0	14.3	1346.7	6.2	7.9	5.1
	*p*	<0.01	<0.01	<0.01	<0.01	<0.01	<0.01	<0.01	<0.01
*T. confusum* adults	*F*	35.4	18.4	7.1	2.2	10.1	6.4	3.4	2.4
	*p*	<0.01	<0.01	<0.01	0.08	<0.01	<0.01	<0.01	<0.01
*T. molitor* larvae	*F*	134.1	13.7	75.9	2.9	40.2	1.5	5.4	0.8
	*p*	<0.01	<0.01	<0.01	0.03	<0.01	0.16	<0.01	0.82
*T. molitor* adults	*F*	936.2	42.7	271.7	4.8	469.3	4.0	21.6	2.6
	*p*	<0.01	<0.01	<0.01	0.01	<0.01	0.01	<0.01	<0.01
*T. granarium* larvae	*F*	555.4	20.1	185.3	7.9	10.7	6.7	10.7	3.2
	*p*	<0.01	<0.01	<0.01	<0.01	<0.01	<0.01	<0.01	<0.01
*T. granarium* adults	*F*	3906.9	62.2	29.0	4.0	849.1	4.1	8.1	4.1
	*p*	<0.01	<0.01	<0.01	0.01	<0.01	0.01	<0.01	<0.01
*O. surinamensis* larvae	*F*	1748.9	105.0	90.1	34.0	692.3	25.2	11.3	5.0
	*p*	<0.01	<0.01	<0.01	<0.01	<0.01	<0.01	<0.01	<0.01
*O. surinamensis* adults	*F*	1362.6	43.5	41.5	3.2	406.0	2.8	8.5	1.1
	*p*	<0.01	<0.01	<0.01	0.02	<0.01	0.01	<0.01	0.28
*R. dominica* adults	*F*	923.3	31.5	28.5	29.9	818.3	37.4	10.3	6.4
	*p*	<0.01	<0.01	<0.01	<0.01	<0.01	<0.01	<0.01	<0.01
*S. oryzae* adults	*F*	1360.4	23.7	232.2	11.7	386.8	8.2	17.5	4.1
	*p*	<0.01	<0.01	<0.01	<0.01	<0.01	<0.01	<0.01	<0.01
*A. siro* nymphs	*F*	9.9	3.9	23.3	1.9	154.1	1.8	3.4	0.6
	*p*	<0.01	0.05	<0.01	0.12	<0.01	0.08	<0.01	0.95
*A. siro* adults	*F*	20.7	5.9	21.3	0.4	475.8	0.7	3.8	0.4
	*p*	<0.01	0.02	<0.01	0.80	<0.01	0.70	<0.01	1.00

## Data Availability

Data are available within the article.

## Supplementary Materials

The following supporting information can be downloaded at: https://www.mdpi.com/article/10.3390/plants14020192/s1, Table S1: Mean (%) mortality ± standard error (SE) of *Alphitobius diaperinus* larvae and adults after 4–16 h, and 1–7 days in wheat treated with *Citrus reticulata*, *Illicium verum*, *Monodora myristica*, and *Xylopia aethiopica* (under the abbreviations *C. r*, *I. v*, *M. m*, and *X. a*, respectively) EOs at two concentrations, and with positive control, pirimiphos-methyl (under the abbreviation P-m); Table S2: Mean (%) mortality ± standard error (SE) of *Tribolium castaneum* larvae and adults after 4–16 h, and 1–7 days in wheat treated with *Citrus reticulata*, *Illicium verum*, *Monodora myristica*, and *Xylopia aethiopica* (under the abbreviations *C. r*, *I. v*, *M. m*, and *X. a*, respectively) EOs at two concentrations, and with positive control, pirimiphos-methyl (under the abbreviation P-m); Table S3: Mean (%) mortality ± standard error (SE) of *Tribolium confusum* larvae and adults after 4–16 h, and 1–7 days in wheat treated with *Citrus reticulata*, *Illicium verum*, *Monodora myristica*, and *Xylopia aethiopica* (under the abbreviations *C. r*, *I. v*, *M. m*, and *X. a*, respectively) EOs at two concentrations, and with positive control, pirimiphos-methyl (under the abbreviation P-m); Table S4: Mean (%) mortality ± standard error (SE) of *Tenebrio molitor* larvae and adults after 4–16 h, and 1–7 days in wheat treated with *Citrus reticulata*, *Illicium verum*, *Monodora myristica*, and *Xylopia aethiopica* (under the abbreviations *C. r*, *I. v*, *M. m*, and *X. a*, respectively) EOs at two concentrations, and with positive control, pirimiphos-methyl (under the abbreviation P-m); Table S5: Mean (%) mortality ± standard error (SE) of *Trogoderma granarium* larvae and adults after 4–16 h, and 1–7 days in wheat treated with *Citrus reticulata*, *Illicium verum*, *Monodora myristica*, and *Xylopia aethiopica* (under the abbreviations *C. r*, *I. v*, *M. m*, and *X. a*, respectively) EOs at two concentrations, and with positive control, pirimiphos-methyl (under the abbreviation P-m); Table S6: Mean (%) mortality ± standard error (SE) of *Oryzaephilus surinamensis* larvae and adults after 4–16 h, and 1–7 days in wheat treated with *Citrus reticulata*, *Illicium verum*, *Monodora myristica*, and *Xylopia aethiopica* (under the abbreviations *C. r*, *I. v*, *M. m*, and *X. a*, respectively) EOs at two concentrations, and with positive control, pirimiphos-methyl (under the abbreviation P-m); Table S7: Mean (%) mortality ± standard error (SE) of *Rhyzopertha dominica* adults after 4–16 h, and 1–7 days in wheat treated with *Citrus reticulata*, *Illicium verum*, *Monodora myristica*, and *Xylopia aethiopica* (under the abbreviations *C. r*, *I. v*, *M. m*, and *X. a*, respectively) EOs at two concentrations, and with positive control, pirimiphos-methyl (under the abbreviation P-m); Table S8: Mean (%) mortality ± standard error (SE) of *Sitophilus oryzae* adults after 4–16 h, and 1–7 days in wheat treated with *Citrus reticulata*, *Illicium verum*, *Monodora myristica*, and *Xylopia aethiopica* (under the abbreviations *C. r*, *I. v*, *M. m*, and *X. a*, respectively) EOs at two concentrations, and with positive control, pirimiphos-methyl (under the abbreviation P-m); Table S9: Mean (%) mortality ± standard error (SE) of *Acarus siro* nymphs and adults after 4–16 h, and 1–7 days in wheat treated with *Citrus reticulata*, *Illicium verum*, *Monodora myristica*, and *Xylopia aethiopica* (under the abbreviations *C. r*, *I. v*, *M. m*, and *X. a*, respectively) EOs at two concentrations, and with positive control, pirimiphos-methyl (under the abbreviation P-m).
