# Supplementary material for: The Potency of Essential Oils in Combating Stored-Product Pests: From Nature to Nemesis
Source: Plants (Basel). 2025 Jan 11;14(2):192. doi: 10.3390/plants14020192 (PMC11768866; doi:10.3390/plants14020192)
Supplement: Supplementary file 1 [file plants-14-00192-s001.zip › plants-3346439-supplementary.pdf]

Table S1. Mean (%) mortality ± standard error (SE) of *Alphitobius diaperinus* larvae and adults after 4–16 h, and 1–7 days in wheat treated with *Citrus reticulata, Illicium verum, Monodora myristica,* and *Xylopia aethiopica* (under the abbreviations *C. r* , *I. v, M. m,* and *X. a*, respectively) EOs at two concentrations, and with positive control, pirimiphos-methyl (under the abbreviation P - m).

**EO type**	***C. r***	***I. v***	***M. m***	***X. a***	**P - m**		
			**Larvae**				
**Exposure**	**Concentration:**	**500 ppm**				***F***	***P***
4 h	0.0 ± 0.0 D	0.0 ± 0.0 C	0.0 ± 0.0 D	0.0 ± 0.0 C	1.1 ± 1.1 E	1.0	0.42
8 h	0.0 ± 0.0 D	0.0 ± 0.0 C	0.0 ± 0.0 D	0.0 ± 0.0 C	2.2 ± 1.5 DE	2.3	0.08
16 h	0.0 ± 0.0 Db	0.0 ± 0.0 Cb	0.0 ± 0.0 Db	0.0 ± 0.0 Cb	5.6 ± 1.8 CDEa	10.0	<0.01
1 d	0.0 ± 0.0 Db	10.0 ± 2.9 Ba	0.0 ± 0.0 Db	0.0 ± 0.0 Cb	10.0 ± 3.3 BCDa	11.4	<0.01
2 d	13.3 ± 2.4 Ca	15.6 ± 3.4 ABa	1.1 ± 1.1 Db	0.0 ± 0.0 Cb	16.7 ± 3.3 ABCa	20.3	<0.01
3 d	30.0 ± 2.9 Ba	17.8 ± 3.6 ABab	10.0 ± 3.3 Cbc	3.3 ± 1.7 BCc	22.2 ± 4.0 ABab	7.9	<0.01
4 d	45.6 ± 4.8 ABa	20.0 ± 3.3 ABa	23.3 ± 5.2 Ba	6.7 ± 2.4 ABb	27.8 ± 3.7 Aa	7.7	<0.01
5 d	55.6 ± 5.3 Aa	22.2 ± 3.2 Aa	35.6 ± 6.0 ABa	8.9 ± 2.6 ABb	30.0 ± 2.4 Aa	12.0	<0.01
6 d	63.3 ± 4.8 Aa	25.6 ± 3.0 Ab	35.6 ± 5.3 Aab	14.4 ± 3.8 Ac	30.0 ± 2.4 Aab	12.9	<0.01
7 d	66.7 ± 4.1 Aa	26.7 ± 3.3 Abc	56.7 ± 4.4 Aab	17.8 ± 4.0 Ac	36.7 ± 5.2 Aab	10.9	<0.01
F	273.8	29.6	62.4	14.2	16.3		
P	<0.01	<0.01	<0.01	<0.01	<0.01		
	Concentration:	1000 ppm					
4 h	0.0 ± 0.0 D	0.0 ± 0.0 F	0.0 ± 0.0 D	0.0 ± 0.0 E	2.2 ± 1.5 E	2.3	0.08
8 h	0.0 ± 0.0 Db	0.0 ± 0.0 Fb	0.0 ± 0.0 Db	0.0 ± 0.0 Eb	3.3 ± 1.7 DEa	4.0	<0.01
16 h	0.0 ± 0.0 Db	0.0 ± 0.0 Fb	0.0 ± 0.0 Db	0.0 ± 0.0 Eb	5.6 ± 1.8 CDEa	10.0	<0.01
1 d	0.0 ± 0.0 Dc	55.6 ± 3.8 Ea	3.3 ± 1.7 Dbc	7.8 ± 1.5 Db	7.8 ± 1.5 BCDb	27.6	<0.01
2 d	27.8 ± 4.0 Cab	68.9 ± 3.1 Da	8.9 ± 2.6 Cc	12.2 ± 2.2 CDbc	13.3 ± 2.9 ABCbc	11.1	<0.01
3 d	65.6 ± 5.8 Ba	75.6 ± 4.4 CDa	24.4 ± 2.9 Bb	18.9 ± 3.1 BCb	18.9 ± 2.6 ABb	31.5	<0.01
4 d	85.6 ± 6.5 ABa	83.3 ± 4.1 BCa	48.9 ± 3.9 ABb	33.3 ± 3.7 ABc	22.2 ± 2.2 ABd	39.7	<0.01
5 d	98.9 ± 1.1 Aa	91.1 ± 3.1 ABa	68.9 ± 3.9 Ab	41.1 ± 3.9 Ac	27.8 ± 2.2 Ad	77.3	<0.01
6 d	100.0 ± 0.0 Aa	98.9 ± 1.1 Aa	83.3 ± 3.3 Aa	47.8 ± 3.6 Ab	33.3 ± 3.3 Ac	68.0	<0.01

7 d	100.0 ± 0.0 Aa	100.0 ± 0.0 Aa	91.1 ± 2.6 Aa	53.3 ± 4.4 Ab	37.8 ± 4.0 Ac	45.7	<0.01
F	1226.1	3259.4	100.2	97.5	17.3		
P	<0.01	<0.01	<0.01	<0.01	<0.01		
			**Adults**				
	Concentration:	500 ppm					
4 h	0.0 ± 0.0	0.0 ± 0.0 B	0.0 ± 0.0	0.0 ± 0.0	0.0 ± 0.0 C	-	-
8 h	0.0 ± 0.0	0.0 ± 0.0 B	0.0 ± 0.0	0.0 ± 0.0	0.0 ± 0.0 C	-	-
16 h	0.0 ± 0.0	0.0 ± 0.0 B	0.0 ± 0.0	0.0 ± 0.0	0.0 ± 0.0 C	-	-
1 d	0.0 ± 0.0	0.0 ± 0.0 B	0.0 ± 0.0	0.0 ± 0.0	0.0 ± 0.0 C	-	-
2 d	0.0 ± 0.0	0.0 ± 0.0 B	0.0 ± 0.0	0.0 ± 0.0	0.0 ± 0.0 C	-	-
3 d	1.1 ± 1.1 ab	0.0 ± 0.0 Ba	0.0 ± 0.0 a	0.0 ± 0.0 a	3.3 ± 1.7 BCa	2.6	0.05
4 d	1.1 ± 1.1 b	8.9 ± 3.9 Aa	0.0 ± 0.0 b	1.1 ± 1.1 b	6.7 ± 2.9 Ba	2.8	0.04
5 d	1.1 ± 1.1 b	8.9 ± 3.9 Aab	0.0 ± 0.0 b	1.1 ± 1.1 bc	16.7 ± 2.9 Aa	10.8	<0.01
6 d	2.2 ± 1.5 bc	8.9 ± 3.9 Ab	0.0 ± 0.0 c	1.1 ± 1.1 bc	20.0 ± 2.4 Aa	14.7	<0.01
7 d	2.2 ± 1.5 bc	8.9 ± 3.9 Ab	0.0 ± 0.0 c	1.1 ± 1.1 bc	22.2 ± 2.2 Aa	16.0	<0.01
F	1.0	4.2	-	0.7	32.9		
P	0.41	<0.01	-	0.74	<0.01		
	Concentration:	1000 ppm					
4 h	0.0 ± 0.0 B	0.0 ± 0.0	0.0 ± 0.0	0.0 ± 0.0 B	0.0 ± 0.0 D	-	-
8 h	0.0 ± 0.0 B	0.0 ± 0.0	0.0 ± 0.0	0.0 ± 0.0 B	0.0 ± 0.0 D	-	-
16 h	0.0 ± 0.0 B	0.0 ± 0.0	0.0 ± 0.0	0.0 ± 0.0 B	0.0 ± 0.0 D	-	-
1 d	0.0 ± 0.0 B	0.0 ± 0.0	0.0 ± 0.0	0.0 ± 0.0 B	0.0 ± 0.0 D	-	-
2 d	1.1 ± 1.1 B	0.0 ± 0.0	0.0 ± 0.0	0.0 ± 0.0 B	0.0 ± 0.0 D	1.0	0.42
3 d	3.3 ± 1.7 AB	0.0 ± 0.0	0.0 ± 0.0	0.0 ± 0.0 B	3.3 ± 2.4 CD	2.4	0.07
4 d	4.4 ± 1.8 AB	4.4 ± 2.9	0.0 ± 0.0	3.3 ± 1.7 A	8.9 ± 4.6 BC	1.5	0.23
5 d	6.7 ± 2.9 ABab	4.4 ± 2.9 b	0.0 ± 0.0 b	4.4 ± 2.4 Aab	17.8 ± 4.0 ABa	4.8	<0.01
6 d	8.9 ± 3.5 ABb	4.4 ± 2.9 b	0.0 ± 0.0 b	4.4 ± 2.4 Ab	24.4 ± 5.0 Aa	9.6	<0.01
7 d	11.1 ± 3.1 Aab	4.4 ± 2.9 bc	0.0 ± 0.0 c	5.6 ± 2.9 Abc	26.7 ± 4.7 Aa	11.4	<0.01
F	4.2	1.5	-	2.6	26.0		
P	0.01	0.15	-	0.01	<0.01		

For each EO, within each row, means followed by the same lowercase letter are not significantly different (df = 9, 89; Tukey HSD test at P = 0.05). For each concentration, within each column, means followed by the same uppercase letter are not significantly different (df = 4, 44; Tukey HSD test at P = 0.05). No significant differences were recorded where no letters exist. No statistical analysis was performed where dashes exist.

Table S2. Mean (%) mortality ± standard error (SE) of *Tribolium castaneum* larvae and adults after 4–16 h, and 1–7 days in wheat treated with *Citrus reticulata, Illicium verum, Monodora myristica,* and *Xylopia aethiopica* (under the abbreviations *C. r* , *I. v, M. m,* and *X. a,* respectively) EOs at two concentrations, and with positive control, pirimiphos-methyl (under the abbreviation P - m).

**EO type**	***C. r***	***I. v***	***M. m***	***X. a***	**P - m**		
			**Larvae**				
**Exposure**	**Concentration:**	**500 ppm**				***F***	***P***
4 h	0.0 ± 0.0 D	0.0 ± 0.0 E	0.0 ± 0.0 D	0.0 ± 0.0 E	0.0 ± 0.0 E	-	-
8 h	0.0 ± 0.0 D	0.0 ± 0.0 E	0.0 ± 0.0 D	0.0 ± 0.0 E	2.2 ± 1.5 E	2.3	0.08
16 h	6.7 ± 2.4 Ca	0.0 ± 0.0 Eb	3.3 ± 1.7 CDab	0.0 ± 0.0 Eb	8.9 ± 2.6 Da	5.7	<0.01
1 d	8.9 ± 2.0 Cab	15.6 ± 2.4 Da	8.9 ± 2.6 BCab	3.3 ± 1.7 Db	21.1 ± 3.1 Ca	5.7	<0.01
2 d	14.4 ± 3.4 BCb	30.0 ± 2.9 Ca	12.2 ± 1.5 ABb	14.4 ± 1.8 Cab	24.4 ± 3.8 BCab	4.5	<0.01
3 d	23.3 ± 4.1 ABbc	36.7 ± 3.3 BCa	13.3 ± 1.7 ABc	32.2 ± 2.8 Bab	30.0 ± 3.3 ABCab	10.6	<0.01
4 d	34.4 ± 5.3 ABb	57.8 ± 3.6 ABa	17.8 ± 2.2 Ac	45.6 ± 1.8 ABab	37.8 ± 3.6 ABCab	16.9	<0.01
5 d	45.6 ± 6.0 Ab	81.1 ± 3.5 Aa	22.2 ± 3.2 Ac	60.0 ± 3.3 ABab	43.3 ± 2.9 ABCb	24.9	<0.01
6 d	51.1 ± 6.1 Ac	84.4 ± 3.8 Aa	24.4 ± 3.4 Ad	78.9 ± 4.2 Aab	53.3 ± 5.0 ABbc	24.1	<0.01
7 d	56.7 ± 5.8 Ac	86.7 ± 4.1 Aa	24.4 ± 3.4 Ad	85.6 ± 4.1 Aab	58.9 ± 4.5 Abc	31.4	<0.01
F	41.0	258.3	35.1	197.7	52.6		
P	<0.01	<0.01	<0.01	<0.01	<0.01		
	Concentration:	1000 ppm					
4 h	3.3 ± 1.7 Ea	0.0 ± 0.0 Db	0.0 ± 0.0 Eb	0.0 ± 0.0 Db	0.0 ± 0.0 Fb	4.0	<0.01
8 h	12.2 ± 3.6 DEa	3.3 ± 1.7 Dab	1.1 ± 1.1 DEb	3.3 ± 2.4 CDab	2.2 ± 1.5 EFab	2.7	0.04
16 h	25.6 ± 4.4 CDa	8.9 ± 2.0 Cab	5.6 ± 2.4 Db	5.6 ± 2.4 Cb	6.7 ± 1.7 DEab	3.3	0.02
1 d	30.0 ± 5.5 BCDab	42.2 ± 4.3 Ba	13.3 ± 2.9 Cc	16.7 ± 3.3 Bbc	16.7 ± 3.3 CDbc	2.7	0.05
2 d	46.7 ± 6.2 ABCab	67.8 ± 5.2 ABa	20.0 ± 2.9 BCbc	35.6 ± 1.8 Aabc	23.3 ± 4.4 BCc	7.4	<0.01
3 d	60.0 ± 6.5 ABCa	77.8 ± 3.2 ABa	27.8 ± 3.2 ABCb	63.3 ± 5.5 Aa	28.9 ± 3.5 ABCb	19.3	<0.01
4 d	71.1 ± 4.6 ABa	96.7 ± 1.7 Aa	33.3 ± 2.9 ABb	80.0 ± 6.0 Aa	34.4 ± 3.8 ABCb	40.0	<0.01
5 d	82.2 ± 2.8 Aa	100.0 ± 0.0 Aa	38.9 ± 3.5 ABb	94.4 ± 2.9 Aa	41.1 ± 2.6 ABb	67.9	<0.01
6 d	93.3 ± 2.4 Aa	100.0 ± 0.0 Aa	46.7 ± 4.1 ABb	98.9 ±1.1 Aa	52.2 ± 3.6 ABb	49.5	<0.01
7 d	100.0 ± 0.0 Aa	100.0 ± 0.0 Aa	52.2 ± 4.0 Ab	100.0 ± 0.0 Aa	60.0 ± 3.7 Ab	48.1	<0.01

F	22.4	93.1	48.7	55.3	34.7		
P	<0.01	<0.01	<0.01	<0.01	<0.01		
			**Adults**				
	Concentration:	500 ppm					
4 h	0.0 ± 0.0	0.0 ± 0.0	0.0 ± 0.0	0.0 ± 0.0	0.0 ± 0.0	-	-
8 h	0.0 ± 0.0	0.0 ± 0.0	0.0 ± 0.0	0.0 ± 0.0	0.0 ± 0.0	-	-
16 h	0.0 ± 0.0	0.0 ± 0.0	0.0 ± 0.0	0.0 ± 0.0	0.0 ± 0.0	-	-
1 d	0.0 ± 0.0	0.0 ± 0.0	0.0 ± 0.0	0.0 ± 0.0	0.0 ± 0.0	-	-
2 d	0.0 ± 0.0	0.0 ± 0.0	0.0 ± 0.0	0.0 ± 0.0	0.0 ± 0.0	-	-
3 d	0.0 ± 0.0	0.0 ± 0.0	0.0 ± 0.0	0.0 ± 0.0	0.0 ± 0.0	-	-
4 d	0.0 ± 0.0	0.0 ± 0.0	0.0 ± 0.0	0.0 ± 0.0	0.0 ± 0.0	-	-
5 d	0.0 ± 0.0	0.0 ± 0.0	0.0 ± 0.0	0.0 ± 0.0	0.0 ± 0.0	-	-
6 d	0.0 ± 0.0	0.0 ± 0.0	0.0 ± 0.0	0.0 ± 0.0	0.0 ± 0.0	-	-
7 d	0.0 ± 0.0	0.0 ± 0.0	0.0 ± 0.0	2.2 ± 2.2	0.0 ± 0.0	1.0	0.42
F	-	-	-	1.0	-		
P	-	-	-	0.45	-		
	Concentration:	1000 ppm					
4 h	0.0 ± 0.0	0.0 ± 0.0	0.0 ± 0.0	0.0 ± 0.0 B	0.0 ± 0.0	-	-
8 h	0.0 ± 0.0	0.0 ± 0.0	0.0 ± 0.0	0.0 ± 0.0 B	0.0 ± 0.0	-	-
16 h	0.0 ± 0.0	0.0 ± 0.0	0.0 ± 0.0	0.0 ± 0.0 B	0.0 ± 0.0	-	-
1 d	0.0 ± 0.0	0.0 ± 0.0	0.0 ± 0.0	0.0 ± 0.0 B	0.0 ± 0.0	-	-
2 d	0.0 ± 0.0	0.0 ± 0.0	0.0 ± 0.0	0.0 ± 0.0 B	0.0 ± 0.0	-	-
3 d	0.0 ± 0.0	0.0 ± 0.0	0.0 ± 0.0	0.0 ± 0.0 B	0.0 ± 0.0	-	-
4 d	0.0 ± 0.0	0.0 ± 0.0	0.0 ± 0.0	0.0 ± 0.0 B	0.0 ± 0.0	-	-
5 d	0.0 ± 0.0	0.0 ± 0.0	0.0 ± 0.0	0.0 ± 0.0 B	0.0 ± 0.0	-	-
6 d	0.0 ± 0.0	0.0 ± 0.0	0.0 ± 0.0	1.1 ±1.1 AB	0.0 ± 0.0	1.0	0.42
7 d	0.0 ± 0.0 b	0.0 ± 0.0 b	0.0 ± 0.0 b	4.4 ± 2.4 Aa	0.0 ± 0.0 b	3.9	<0.01
F		-	-	2.9	-		
P		-	-	<0.01	-		

For each EO, within each row, means followed by the same lowercase letter are not significantly different (df = 9, 89; Tukey HSD test at P = 0.05). For each concentration, within each column, means followed by the same uppercase letter are not significantly different (df = 4, 44; Tukey HSD test at P = 0.05). No significant differences were recorded where no letters exist. No statistical analysis was performed where dashes exist.

Table S3. Mean (%) mortality ± standard error (SE) of *Tribolium confusum* larvae and adults after 4–16 h, and 1–7 days in wheat treated with *Citrus reticulata, Illicium verum, Monodora myristica,* and *Xylopia aethiopica* (under the abbreviations *C. r*, *I. v*, *M. m*, and *X. a*, respectively) EOs at two concentrations, and with positive control, pirimiphos-methyl (under the abbreviation P - m).

**EO type**	***C. r***	***I. v***	***M. m***	***X. a***	**P - m**		
			**Larvae**				
**Exposure**	**Concentration:**	**500 ppm**				***F***	***P***
4 h	0.0 ± 0.0 F	0.0 ± 0.0 D	0.0 ± 0.0 B	0.0 ± 0.0 C	0.0 ± 0.0 E	-	-
8 h	0.0 ± 0.0 F	0.0 ± 0.0 D	0.0 ± 0.0 B	0.0 ± 0.0 C	2.2 ± 1.5 E	2.3	0.08
16 h	4.4 ± 1.8 EFa	0.0 ± 0.0 Db	0.0 ± 0.0 Bb	0.0 ± 0.0 Cb	6.7 ± 1.7 Da	8.4	<0.01
1 d	6.7 ± 1.7 DEa	0.0 ± 0.0 Db	0.0 ± 0.0 Bb	4.4 ± 2.4 BCab	13.3 ± 3.3 CDa	8.8	<0.01
2 d	7.8 ± 2.2 CDEa	15.6 ± 3.8 Ca	0.0 ± 0.0 Bb	11.7 ± 3.9 Ba	23.3 ± 2.9 BCa	9.7	<0.01
3 d	14.4 ± 4.4 BCDEb	35.6 ± 5.6 Ba	2.2 ± 1.5 ABc	18.9 ± 3.5 Aab	31.1 ± 2.6 ABa	18.3	<0.01
4 d	23.3 ± 6.0 ABCDb	62.2 ± 8.0 ABa	4.4 ± 2.4 ABc	24.4 ± 4.4 Aab	44.4 ± 3.8 ABab	18.0	<0.01
5 d	32.2 ± 6.4 ABCa	76.7 ± 9.4 ABa	6.7 ± 2.4 ABb	31.1 ± 4.8 Aa	55.6 ± 4.8 ABa	13.4	<0.01
6 d	41.1 ± 5.4 ABab	87.8 ± 4.9 Aa	8.9 ± 2.6 Ac	35.6 ± 4.7 Ab	62.2 ± 4.3 ABab	22.3	<0.01
7 d	44.4 ± 5.8 Aa	91.1 ± 4.2 Aa	10.0 ± 2.9 Ab	42.2 ± 5.7 Aa	66.7 ± 4.7 Aa	20.5	<0.01
F	17.6	120.8	6.3	39.6	46.3		
P	<0.01	<0.01	<0.01	<0.01	<0.01		
	Concentration:	1000 ppm					
4 h	0.0 ± 0.0 E	0.0 ± 0.0 E	0.0 ± 0.0 E	0.0 ± 0.0 D	0.0 ± 0.0 E	-	-
8 h	0.0 ± 0.0 Eb	0.0 ± 0.0 Eb	4.4 ± 1.8 DEa	0.0 ± 0.0 Db	3.3 ± 1.7 Eab	4.0	<0.01
16 h	4.4 ± 1.8 DE	4.4 ± 1.8 D	7.8 ± 2.2 CD	2.2 ± 1.5 CD	8.9 ± 2.0 D	1.9	0.12
1 d	12.2 ± 3.6 CD	21.1 ± 5.4 C	16.7 ± 3.3 BC	6.7 ± 3.3 C	14.4 ± 3.4 CD	2.4	0.07
2 d	18.9 ± 3.9 BC	35.6 ± 4.8 BC	24.4± 4.4 AB	20.0 ± 3.3 B	23.3 ± 3.7 BCD	1.1	0.35
3 d	37.8 ± 6.2 ABb	63.3 ± 5.8 ABa	34.4 ± 3.8 ABb	34.4 ± 2.4 ABb	30.0 ± 2.4 ABCb	7.2	<0.01
4 d	52.2 ± 7.4 Ab	87.8 ± 4.0 Aa	42.2 ± 4.3 Ab	45.6 ± 4.1 ABb	42.2 ± 3.2 ABb	10.3	<0.01
5 d	72.2 ± 6.4 Ab	98.9 ± 1.1 Aa	50.0 ± 3.3 Ac	58.9 ± 4.6 Abc	56.7 ± 4.7 Abc	13.8	<0.01
6 d	85.6 ± 5.8 Aa	100.0 ± 0.0 Aa	55.6 ± 2.9 Ab	64.4 ±3.4 Ab	63.3 ± 3.3 Ab	21.0	<0.01

7 d	97.8 ± 1.5 Aa	100.0 ± 0.0 Aa	62.2 ± 3.2 Ac	76.7 ± 3.7 Ab	66.7 ± 2.4 Abc	7.4	<0.01
F	59.1	100.8	36.6	63.7	37.2		
P	<0.01	<0.01	<0.01	<0.01	<0.01		
			**Adults**				
	Concentration:	500 ppm					
4 h	0.0 ± 0.0	0.0 ± 0.0	0.0 ± 0.0	0.0 ± 0.0 B	0.0 ± 0.0	-	-
8 h	0.0 ± 0.0	0.0 ± 0.0	0.0 ± 0.0	0.0 ± 0.0 B	0.0 ± 0.0	-	-
16 h	0.0 ± 0.0	0.0 ± 0.0	0.0 ± 0.0	0.0 ± 0.0 B	0.0 ± 0.0	-	-
1 d	0.0 ± 0.0	0.0 ± 0.0	0.0 ± 0.0	0.0 ± 0.0 B	0.0 ± 0.0	-	-
2 d	0.0 ± 0.0	0.0 ± 0.0	0.0 ± 0.0	0.0 ± 0.0 B	0.0 ± 0.0	-	-
3 d	0.0 ± 0.0	0.0 ± 0.0	0.0 ± 0.0	1.1 ± 1.1 AB	0.0 ± 0.0		
4 d	0.0 ± 0.0	0.0 ± 0.0	0.0 ± 0.0	2.2 ± 1.5 AB	0.0 ± 0.0		
5 d	0.0 ± 0.0 b	0.0 ± 0.0 b	0.0 ± 0.0 b	4.4 ± 2.4 Aa	0.0 ± 0.0 b		
6 d	0.0 ± 0.0 b	0.0 ± 0.0 b	0.0 ± 0.0 b	5.6 ± 2.4 Aa	0.0 ± 0.0 b		
7 d	0.0 ± 0.0 b	0.0 ± 0.0 b	0.0 ± 0.0 b	5.6 ± 2.4 Aa	0.0 ± 0.0 b		
F	-	-	-	3.0	-		
P	-	-	-	<0.01	-		
	Concentration:	1000 ppm					
4 h	0.0 ± 0.0	0.0 ± 0.0 B	0.0 ± 0.0 B	0.0 ± 0.0 C	0.0 ± 0.0	-	-
8 h	0.0 ± 0.0	0.0 ± 0.0 B	0.0 ± 0.0 B	0.0 ± 0.0 C	0.0 ± 0.0	-	-
16 h	0.0 ± 0.0	0.0 ± 0.0 B	0.0 ± 0.0 B	0.0 ± 0.0 C	0.0 ± 0.0	-	-
1 d	0.0 ± 0.0 b	4.4 ± 1.8 ABa	0.0 ± 0.0 Bb	3.3 ± 1.7 BCab	0.0 ± 0.0 b	4.0	<0.01
2 d	0.0 ± 0.0 b	5.6 ± 2.4 Aa	0.0 ± 0.0 Bb	3.3 ± 1.7 BCab	0.0 ± 0.0 b	4.0	<0.01
3 d	0.0 ± 0.0 b	5.6 ± 2.4 Aa	1.1 ± 1.1 ABab	6.7 ± 2.9 ABCa	0.0 ± 0.0 b	3.6	0.01
4 d	0.0 ± 0.0 b	5.6 ± 2.4 Aab	1.1 ± 1.1 ABb	10.0 ± 2.9 ABa	0.0 ± 0.0 b	6.6	<0.01
5 d	0.0 ± 0.0 b	5.6 ± 2.4 Aab	3.3 ± 1.7 ABb	13.3 ± 3.3 ABa	0.0 ± 0.0 b	7.5	<0.01
6 d	1.1 ± 1.1 b	5.6 ± 2.4 Aab	4.4 ± 1.8 Aab	15.6 ±4.1 ABa	0.0 ± 0.0 b	6.1	<0.01
7 d	2.2 ± 1.5 b	5.6 ± 2.4 Ab	5.6 ± 2.4 Ab	18.9 ± 4.6 Aa	0.0 ± 0.0 b	7.4	<0.01
F	1.7	2.1	3.2	8.0	-		
P	0.11	0.04	<0.01	<0.01	-		

For each EO, within each row, means followed by the same lowercase letter are not significantly different (df = 9, 89; Tukey HSD test at P = 0.05). For each concentration, within each column, means followed by the same uppercase letter are not significantly different (df = 4, 44; Tukey HSD test at P = 0.05). No significant differences were recorded where no letters exist. No statistical analysis was performed where dashes exist.

Table S4. Mean (%) mortality ± standard error (SE) of *Tenebrio molitor* larvae and adults after 4–16 h, and 1–7 days in wheat treated with *Citrus reticulata, Illicium verum, Monodora myristica,* and *Xylopia aethiopica* (under the abbreviations *C. r*, *I. v*, *M. m*, and *X. a*, respectively) EOs at two concentrations, and with positive control, pirimiphos-methyl (under the abbreviation P - m).

**EO type**	***C. r***	***I. v***	***M. m***	***X. a***	**P - m**		
			**Larvae**				
**Exposure**	**Concentration:**	**500 ppm**				***F***	***P***
4 h	0.0 ± 0.0	0.0 ± 0.0 B	0.0 ± 0.0	0.0 ± 0.0	0.0 ± 0.0 E	-	-
8 h	0.0 ± 0.0	0.0 ± 0.0 B	0.0 ± 0.0	0.0 ± 0.0	0.0 ± 0.0 E	-	-
16 h	0.0 ± 0.0	0.0 ± 0.0 B	0.0 ± 0.0	0.0 ± 0.0	0.0 ± 0.0 E	-	-
1 d	0.0 ± 0.0	0.0 ± 0.0 B	0.0 ± 0.0	0.0 ± 0.0	1.1 ± 1.1 E	1.0	0.42
2 d	0.0 ± 0.0 b	0.0 ± 0.0 Bb	0.0 ± 0.0 b	1.1 ± 1.1 ab	3.3 ± 1.7 DEa	2.6	0.05
3 d	0.0 ± 0.0 b	0.0 ± 0.0 Bb	0.0 ± 0.0 b	1.1 ± 1.1 b	6.7 ± 1.7 CDa	10.5	<0.01
4 d	0.0 ± 0.0 b	2.2 ± 1.5 ABb	0.0 ± 0.0 b	1.1 ± 1.1 b	12.2 ± 2.8 BCa	16.0	<0.01
5 d	0.0 ± 0.0 c	4.4 ± 1.8 ABb	0.0 ± 0.0 c	1.1 ± 1.1 bc	15.6 ± 2.9 ABa	24.0	<0.01
6 d	0.0 ± 0.0 c	5.6 ± 1.8 Ab	0.0 ± 0.0 c	3.3 ± 1.7 bc	22.2 ± 4.7 ABa	20.2	<0.01
7 d	0.0 ± 0.0 c	6.7 ± 2.4 Ab	0.0 ± 0.0 c	3.3 ± 1.7 bc	31.1 ± 3.5 Aa	26.8	<0.01
F	-	5.4	-	1.6	32.8		
P	-	<0.01	-	0.14	<0.01		
	Concentration:	1000 ppm					
4 h	0.0 ± 0.0 B	0.0 ± 0.0 B	0.0 ± 0.0	0.0 ± 0.0 C	0.0 ± 0.0 E	-	-
8 h	0.0 ± 0.0 B	0.0 ± 0.0 B	0.0 ± 0.0	0.0 ± 0.0 C	0.0 ± 0.0 E	-	-
16 h	0.0 ± 0.0 B	0.0 ± 0.0 B	0.0 ± 0.0	0.0 ± 0.0 C	0.0 ± 0.0 E	-	-
1 d	2.2 ± 1.5 AB	3.3 ± 1.7 AB	0.0 ± 0.0	1.1 ± 1.1 BC	2.2 ± 1.5 DE	1.0	0.44
2 d	3.3 ± 1.7 AB	3.3 ± 1.7 AB	0.0 ± 0.0	3.3 ± 1.7 ABC	4.4 ± 1.8 DE	1.2	0.31
3 d	5.6 ± 2.4 AB	5.6 ± 2.9 AB	0.0 ± 0.0	3.3 ± 1.7 ABC	5.6 ± 1.8 CDE	1.7	0.17
4 d	7.8 ± 2.8 ABab	7.8 ± 2.8 ABab	0.0 ± 0.0 b	3.3 ± 1.7 ABCab	7.8 ± 1.5 BCDa	3.7	0.01
5 d	10.0 ± 2.9 Aa	8.9 ± 2.6 Aa	0.0 ± 0.0 b	6.7 ± 3.3 ABCab	15.6 ± 3.4 ABCa	5.8	<0.01
6 d	10.0 ± 2.9 Aa	11.1 ± 2.6 Aa	0.0 ± 0.0 b	10.0 ±4.1 ABa	20.0 ± 3.3 ABa	7.3	<0.01

7 d	10.0 ± 2.9 Ab	15.6 ± 3.8 Aab	0.0 ± 0.0 c	14.4 ± 4.4 Aab	32.2 ± 2.8 Aa	12.7	<0.01
F	4.5	5.6	-	4.9	18.5		
P	<0.01	<0.01	-	<0.01	<0.01		
			**Adults**				
	Concentration:	500 ppm					
4 h	0.0 ± 0.0 C	0.0 ± 0.0 B	0.0 ± 0.0 B	0.0 ± 0.0 C	0.0 ± 0.0 E	-	-
8 h	0.0 ± 0.0 C	0.0 ± 0.0 B	0.0 ± 0.0 B	0.0 ± 0.0 C	0.0 ± 0.0 E	-	-
16 h	0.0 ± 0.0 C	0.0 ± 0.0 B	0.0 ± 0.0 B	0.0 ± 0.0 C	1.1 ± 1.1 E	1.0	0.42
1 d	0.0 ± 0.0 Cb	0.0 ± 0.0 Bb	0.0 ± 0.0 Bb	1.1 ± 1.1 Cb	8.9 ± 2.0 Da	17.0	<0.01
2 d	0.0 ± 0.0 Cb	0.0 ± 0.0 Bb	2.2 ± 1.5 Bb	7.8 ± 4.7 BCb	13.3 ± 2.9 CDa	9.8	<0.01
3 d	3.3 ± 1.7 Cbc	0.0 ± 0.0 Bc	4.4 ± 2.4 ABbc	17.8 ± 6.6 ABab	22.2 ± 3.2 BCa	10.7	<0.01
4 d	13.3 ± 2.9 Bab	2.2 ± 1.5 ABc	5.6 ± 2.4 ABbc	22.2 ± 6.8 ABab	31.1 ± 3.1 ABa	9.5	<0.01
5 d	25.6 ± 3.4 ABa	3.3 ± 1.7 ABc	7.8 ± 3.2 ABbc	24.4 ± 7.5 ABab	41.1 ± 4.2 ABa	10.5	<0.01
6 d	35.6 ± 4.4 Aa	5.6 ± 2.4 ABb	8.9 ± 3.9 ABb	32.2 ± 6.4 Aa	53.3 ± 3.7 Aa	15.8	<0.01
7 d	47.8 ± 5.2 Aa	7.8 ± 2.8 Ab	13.3 ± 3.7 Ab	37.8 ± 6.2 Aa	66.7 ± 3.7 Aa	14.9	<0.01
F	84.6	4.4	4.5	16.6	75.4		
P	<0.01	<0.01	<0.01	<0.01	<0.01		
	Concentration:	1000 ppm					
4 h	0.0 ± 0.0 E	0.0 ± 0.0 C	0.0 ± 0.0 D	0.0 ± 0.0 C	0.0 ± 0.0 E	-	-
8 h	0.0 ± 0.0 E	0.0 ± 0.0 C	0.0 ± 0.0 D	0.0 ± 0.0 C	0.0 ± 0.0 E	-	-
16 h	0.0 ± 0.0 E	0.0 ± 0.0 C	0.0 ± 0.0 D	0.0 ± 0.0 C	1.1 ± 1.1 E	1.0	0.42
1 d	3.3 ± 1.7 DEbc	0.0 ± 0.0 Cc	0.0 ± 0.0 Dc	22.2 ± 4.7 Ba	8.9 ± 2.0 Dab	17.0	<0.01
2 d	5.6 ± 1.8 Db	3.3 ± 1.7 BCb	5.6 ± 1.8 Cb	47.8 ± 8.3 ABa	12.2 ± 2.8 CDab	8.4	<0.01
3 d	15.6 ± 2.9 Cbc	5.6 ± 1.8 ABCc	12.2 ± 2.8 BCbc	65.6 ± 10.6 Aa	23.3 ± 4.1 BCab	8.8	<0.01
4 d	26.7 ± 2.4 BCab	8.9 ± 2.0 ABc	15.6 ± 2.9 ABbc	71.7 ± 11.2 Aa	34.4 ± 2.9 ABab	10.5	<0.01
5 d	52.2 ± 5.2 ABa	11.1 ± 2.6 ABc	20.0 ± 3.3 ABbc	76.7 ± 11.7 Aa	43.3 ± 2.4 ABab	13.7	<0.01
6 d	68.9 ± 6.3 ABa	14.4 ± 3.4 ABc	24.4 ± 2.9 ABbc	81.1 ±11.1 Aa	55.6 ± 2.9 Aab	12.6	<0.01
7 d	78.9 ± 7.2 Aa	16.7 ± 4.4 Ab	32.2 ± 3.2 Aa	83.3 ± 10.3 Aa	67.8 ± 1.5 Aa	13.3	<0.01
F	67.2	10.1	40.3	72.2	66.3		
P	<0.01	<0.01	<0.01	<0.01	<0.01		

For each EO, within each row, means followed by the same lowercase letter are not significantly different (df = 9, 89; Tukey HSD test at P = 0.05). For each concentration, within each column, means followed by the same uppercase letter are not significantly different (df = 4, 44; Tukey HSD test at P = 0.05). No significant differences were recorded where no letters exist. No statistical analysis was performed where dashes exist.

Table S5. Mean (%) mortality ± standard error (SE) of *Trogoderma granarium* larvae and adults after 4–16 h, and 1–7 days in wheat treated with *Citrus reticulata, Illicium verum, Monodora myristica,* and *Xylopia aethiopica* (under the abbreviations *C. r*, *I. v*, *M. m*, and *X. a*, respectively) EOs at two concentrations, and with positive control, pirimiphos-methyl (under the abbreviation P - m).

**EO type**	***C. r***	***I. v***	***M. m***	***X. a***	**P - m**		
			**Larvae**				
**Exposure**	**Concentration:**	**500 ppm**				***F***	***P***
4 h	0.0 ± 0.0	0.0 ± 0.0 D	0.0 ± 0.0 B	0.0 ± 0.0	0.0 ± 0.0 D	-	-
8 h	0.0 ± 0.0	0.0 ± 0.0 D	0.0 ± 0.0 B	0.0 ± 0.0	0.0 ± 0.0 D	-	-
16 h	0.0 ± 0.0	0.0 ± 0.0 D	6.7 ± 1.7 A	0.0 ± 0.0	0.0 ± 0.0 D	2.3	0.08
1 d	0.0 ± 0.0 b	0.0 ± 0.0 Db	11.1 ± 2.0 Aa	0.0 ± 0.0 b	2.2 ± 1.5 CDb	22.7	<0.01
2 d	0.0 ± 0.0 b	0.0 ± 0.0 Db	14.4 ± 2.9 Aa	0.0 ± 0.0 b	2.2 ± 1.5 CDb	24.1	<0.01
3 d	0.0 ± 0.0 b	8.9 ± 2.6 Ca	15.6± 2.9 Aa	0.0 ± 0.0 b	5.6 ± 1.8 BCa	12.6	<0.01
4 d	0.0 ± 0.0 b	13.3 ± 3.3 BCa	17.8 ± 2.8 Aa	0.0 ± 0.0 b	13.3 ± 2.9 ABa	21.6	<0.01
5 d	0.0 ± 0.0 b	18.9 ± 3.1 ABa	20.0 ± 3.7 Aa	0.0 ± 0.0 b	18.9 ± 2.6 Aa	70.9	<0.01
6 d	0.0 ± 0.0 b	23.3 ± 2.9 ABa	22.2 ± 4.0 Aa	0.0 ± 0.0 b	22.2 ± 2.22 Aa	81.7	<0.01
7 d	0.0 ± 0.0 b	30.0 ± 4.1 Aa	23.3 ± 4.1 Aa	0.0 ± 0.0 b	25.6 ± 2.4 A	82.0	<0.01
F	-	48.9	12.3	-	32.6		
P	-	<0.01	<0.01	-	<0.01		
	Concentration:	1000 ppm					
4 h	0.0 ± 0.0 D	0.0 ± 0.0 B	0.0 ± 0.0 E	0.0 ± 0.0 D	0.0 ± 0.0 D	-	-
8 h	2.2 ± 1.5 CD	0.0 ± 0.0 B	0.0 ± 0.0 E	0.0 ± 0.0 D	0.0 ± 0.0 D	2.3	0.08
16 h	4.4 ± 1.8 BCDa	0.0 ± 0.0 Bb	3.3 ± 1.7 DEab	0.0 ± 0.0 Db	0.0 ± 0.0 Db	4.0	<0.01
1 d	5.6 ± 1.8 ABCDab	0.0 ± 0.0 Bc	7.8 ± 2.2 CDa	0.0 ± 0.0 Dc	1.1 ± 1.1 CDbc	7.3	<0.01
2 d	7.8 ± 2.2 ABCab	3.3 ± 1.7 Babc	10 ± 2.4 BCDa	0.0 ± 0.0 Dc	2.2 ± 1.5 CDbc	5.5	<0.01
3 d	8.9 ± 2.0 ABab	22.2 ± 4.0 Aa	10 ± 2.4 BCDab	0.0 ± 0.0 Dc	7.8 ± 3.2 BCbc	8.1	<0.01
4 d	10.0 ± 1.7 ABa	30.0 ± 5.3 Aa	14.4 ± 3.8 ABCa	2.2 ± 1.5 Cb	14.4 ± 3.4 ABa	6.7	<0.01
5 d	11.1 ± 1.1 Ab	36.7 ± 5.5 Aa	18.9 ± 3.5 ABCab	3.3 ± 1.7 Bc	17.8 ± 2.8 Aab	21.5	<0.01
6 d	13.3 ± 2.4 Ab	38.9 ± 4.8 Aa	25.6 ± 4.1 ABab	4.4 ± 1.8 Ac	22.2 ± 3.6 Aab	18.2	<0.01
7 d	15.6 ± 2.9 Ab	47.8 ± 5.2 Aa	31.1 ± 3.5 Aab	5.6 ± 2.4 Ac	26.7 ± 2.9 Aab	20.6	<0.01

F	9.0	58.8	16.4	3.5	29.9		
P	<0.01	<0.01	<0.01	<0.01	<0.01		
			**Adults**				
	Concentration:	500 ppm					
4 h	0.0 ± 0.0 D	0.0 ± 0.0 C	0.0 ± 0.0 E	0.0 ± 0.0 C	1.1 ± 1.1 E	1.0	0.42
8 h	0.0 ± 0.0 D	0.0 ± 0.0 C	0.0 ± 0.0 E	0.0 ± 0.0 C	2.2 ± 1.5 E	2.3	0.08
16 h	0.0 ± 0.0 Db	0.0 ± 0.0 Cb	0.0 ± 0.0 Eb	0.0 ± 0.0 Cb	6.7 ± 1.7 Da	16.0	<0.01
1 d	3.3 ± 1.7 CDbc	0.0 ± 0.0 Cc	6.7 ± 1.7 Dab	5.6 ± 1.8 Babc	12.2 ± 2.2 CDa	6.5	<0.01
2 d	4.4 ± 2.4 CDb	2.2 ± 1.0 Cb	23.3 ± 1.7 Ca	31.1 ± 5.4 Aa	20.0 ± 2.9 BCa	24.6	<0.01
3 d	8.9 ± 2.6 BCb	10.0 ± 2.9 Bb	37.8 ± 2.8 BCa	45.6 ± 6.7 Aa	33.3 ± 3.3 ABa	11.0	<0.01
4 d	15.6 ± 3.8 ABCb	22.2 ± 13.8 Aab	48.9 ± 2.6 ABa	52.2 ± 8.8 Aa	42.2 ± 4.3 ABa	6.2	<0.01
5 d	25.6 ± 5.0 ABb	31.1 ± 2.6 Aab	60.0 ± 6.0 ABa	60.0 ± 7.3 Aa	53.3 ± 3.7 ABa	6.3	<0.01
6 d	33.3 ± 6.2 ABb	42.2 ± 2.2 Aab	72.2 ± 6.6 ABa	64.4 ± 7.5 Aa	62.2 ± 3.6 Aa	4.6	<0.01
7 d	36.7 ± 5.8 Ab	42.2 ± 2.2 Ab	80.0 ± 6.0 Aa	67.8 ± 7.0 Aa	71.1 ± 3.9 Aa	11.9	<0.01
F	17.2	58.6	182.2	104.0	43.7		
P	<0.01	<0.01	<0.01	<0.01	<0.01		
	Concentration:	1000 ppm					
4 h	0.0 ± 0.0 D	0.0 ± 0.0 E	0.0 ± 0.0 E	0.0 ± 0.0 D	2.2 ± 1.5 D	2.3	0.08
8 h	0.0 ± 0.0 D	0.0 ± 0.0 E	1.1 ± 1.1 E	0.0 ± 0.0 D	3.3 ± 2.4 CD	1.5	0.21
16 h	0.0 ± 0.0 Dc	3.3 ± 1.7 Dbc	10.0 ± 1.7 Da	0.0 ± 0.0 Dc	7.8 ± 3.2 CDab	8.6	<0.01
1 d	3.3 ± 1.7 Db	21.1 ± 2.6 Ca	24.4 ± 1.0 Ca	11.1 ± 2.0 Ca	13.3 ± 4.7 BCab	8.9	<0.01
2 d	14.4 ± 3.8 Cc	31.1 ± 3.1 BCab	54.4 ± 3.8 Ba	47.8 ± 4.3 Bab	22.2 ± 4.9 ABbc	9.7	<0.01
3 d	33.3 ± 4.4 Bb	60.0 ± 5.8 ABa	68.9 ± 4.5 ABa	66.7 ± 8.0 ABa	34.4 ± 3.8 Ab	9.8	<0.01
4 d	50.0 ± 6.9 ABb	75.6 ± 6.7 Aa	84.4 ± 4.7 ABa	75.6 ± 6.7 ABa	44.4 ± 4.7 Ab	8.8	<0.01
5 d	63.3 ± 6.0 ABb	84.4 ± 5.8 Aa	95.5 ± 2.4 ABa	87.8 ± 3.6 Aa	56.7 ± 4.7 Ab	11.5	<0.01
6 d	80.0 ± 6.5 ABbc	88.9 ± 4.8 Aab	100.0 ± 0.0 Aa	91.1 ± 3.5 Aab	67.8 ± 4.9 Ac	7.2	<0.01
7 d	84.4 ± 5.0 Aab	90.0 ± 4.7 Aab	100.0 ± 0.0 Aa	93.3 ± 2.9 Aa	73.3 ± 6.0 Ab	5.4	<0.01
F	89.1	163.8	189.1	332.8	25.2		
P	<0.01	<0.01	<0.01	<0.01	<0.01		

For each EO, within each row, means followed by the same lowercase letter are not significantly different (df = 9, 89; Tukey HSD test at P = 0.05). For each concentration, within each column, means followed by the same uppercase letter are not significantly different (df = 4, 44; Tukey HSD test at P = 0.05). No significant differences were recorded where no letters exist. No statistical analysis was performed where dashes exist.

Table S6. Mean (%) mortality ± standard error (SE) of *Oryzaephilus surinamensis* larvae and adults after 4–16 h, and 1–7 days in wheat treated with *Citrus reticulata, Illicium verum, Monodora myristica,* and *Xylopia aethiopica* (under the abbreviations *C. r* , *I. v*, *M. m*, and *X. a*, respectively) EOs at two concentrations, and with positive control, pirimiphos-methyl (under the abbreviation P - m).

**EO type**	***C. r***	***I. v***	***M. m***	***X. a***	**P - m**		
			**Larvae**				
**Exposure**	**Concentration:**	**500 ppm**				***F***	***P***
4 h	0.0 ± 0.0 E	0.0 ± 0.0 D	0.0 ± 0.0 C	0.0 ± 0.0 B	0.0 ± 0.0 E	-	-
8 h	0.0 ± 0.0 E	0.0 ± 0.0 D	0.0 ± 0.0 C	0.0 ± 0.0 B	0.0 ± 0.0 E	-	-
16 h	0.0 ± 0.0 E	0.0 ± 0.0 D	0.0 ± 0.0 C	0.0 ± 0.0 B	0.0 ± 0.0 E	-	-
1 d	4.4 ± 1.8 D	2.2 ± 1.5 CD	1.1 ± 1.1 C	0.0 ± 0.0 B	2.2± 1.5 E	1.6	0.20
2 d	7.8 ± 2.2 CDa	5.6 ± 1.8 BCab	2.2 ± 1.5 BCb	2.2 ± 1.5 ABb	8.9 ± 2.0 Da	2.8	0.04
3 d	11.1 ± 2.0 BCab	13.3 ± 2.4 ABa	4.4 ± 1.8 ABCbc	2.2 ± 1.5 ABc	15.6 ± 1.8 Ca	9.0	<0.01
4 d	20.0 ± 2.9 ABab	15.6 ± 2.9 Aab	8.9 ± 2.6 ABbc	4.4 ± 1.8 ABc	24.4 ± 2.4 BCa	7.6	<0.01
5 d	24.4 ± 2.9 ABab	20.0 ± 2.9 Aab	11.1 ± 2.6 Abc	7.8 ± 3.2 ABc	36.7 ± 3.7 ABa	7.3	<0.01
6 d	28.9 ± 2.6 Aab	25.6 ± 2.4 Aab	13.3 ± 2.4 Abc	12.2 ± 4.3 Ac	46.7 ± 5.3 ABa	7.8	<0.01
7 d	35.6 ± 3.4 Aa	28.9 ± 2.0 Aab	13.3 ± 2.4 Abc	12.2 ± 4.3 Ac	63.3 ± 4.7 Aa	11.3	<0.01
F	42.7	29.6	11.2	5.0	93.3		
P	<0.01	<0.01	<0.01	<0.01	<0.01		
	Concentration:	1000 ppm					
4 h	0.0 ± 0.0 D	0.0 ± 0.0 D	0.0 ± 0.0 D	0.0 ± 0.0 E	0.0 ± 0.0 D	-	-
8 h	0.0 ± 0.0 Db	3.3 ± 1.7 CDa	0.0 ± 0.0 Db	0.0 ± 0.0 Eb	0.0 ± 0.0 Db	4.0	<0.01
16 h	1.1 ± 1.1 Db	7.8 ± 2.2 Ca	3.3 ± 1.7 Dab	1.1 ± 1.1 DEb	0.0 ± 0.0 Db	4.6	<0.01
1 d	6.7 ± 1.7 Cb	31.1 ± 4.6 Ba	10.0 ± 2.4 Cab	7.8 ± 2.8 CDb	3.3 ± 1.7 Db	6.5	<0.01
2 d	7.8 ± 1.5 Cb	42.2 ± 4.7 ABa	15.6 ± 2.9 BCab	18.9 ±4.2 BCab	10.0 ± 2.4 Cb	4.2	<0.01
3 d	21.1 ± 2.6 Bab	55.6 ± 5.3 ABa	21.1 ± 2.6 ABCab	31.1 ± 4.8 ABab	15.6 ± 2.4 BCb	4.3	<0.01
4 d	34.4 ± 3.4 ABbc	63.3 ± 6.5 ABa	26.7 ± 3.7 ABc	43.3 ± 5.0 ABab	27.8 ± 2.8 ABbc	9.0	<0.01
5 d	51.1 ± 4.2 ABab	76.7 ± 5.8 Aa	30.0 ± 5.5 ABc	51.1 ± 6.1 Aab	31.1 ± 2.0 ABbc	12.0	<0.01
6 d	58.9 ± 4.8 Aab	85.6 ±5.6 Aa	35.6 ± 5.3 ABc	58.9 ±7.4 Aab	44.4 ± 3.8 Abc	9.6	<0.01
7 d	64.4 ± 5.0 Aab	92.2 ± 3.6 Aa	37.8 ± 4.7 Ac	64.4 ± 7.8 Ab	64.4 ± 3.8 Aab	11.3	<0.01

F	73.3	68.0	38.5	38.8	63.4		
P	<0.01	<0.01	<0.01	<0.01	<0.01		
			**Adults**				
	Concentration:	500 ppm					
4 h	0.0 ± 0.0 D	0.0 ± 0.0 D	0.0 ± 0.0 D	0.0 ± 0.0 C	0.0 ± 0.0 C	-	-
8 h	0.0 ± 0.0 D	0.0 ± 0.0 D	0.0 ± 0.0 D	0.0 ± 0.0 C	0.0 ± 0.0 C	-	-
16 h	0.0 ± 0.0 D	0.0 ± 0.0 D	0.0 ± 0.0 D	0.0 ± 0.0 C	1.1 ± 1.1 BC	1.0	0.42
1 d	0.0 ± 0.0 D	3.3 ± 1.7 C	0.0 ± 0.0 D	1.1 ± 1.1 BC	1.1 ± 1.1 BC	1.8	0.16
2 d	0.0 ± 0.0 Db	26.7 ± 1.7 Ba	1.1 ± 1.1 CDbc	6.7 ± 2.4 Bb	2.2 ± 1.5 ABCbc	22.4	<0.01
3 d	5.6 ± 2.4 Cb	38.9 ± 2.6 ABa	2.2 ± 1.5 BCDb	21.1 ± 4.6 Aa	2.2 ± 1.5 ABCb	16.6	<0.01
4 d	13.3 ± 3.3 Bbc	45.6 ± 4.4 ABa	4.4 ± 1.8 ABCDc	31.1 ± 6.3 Aab	6.7 ± 1.7 ABbc	8.6	<0.01
5 d	23.3 ± 4.1ABa	48.9 ± 3.9 ABa	6.7 ± 2.4 ABCb	37.8 ± 6.4 Aa	7.8 ± 2.2 ABb	13.1	<0.01
6 d	26.7 ± 4.4 ABa	53.3 ± 4.7 Aa	7.8 ± 2.2 ABb	45.6 ± 6.7 Aa	10.0 ± 2.4 Ab	12.6	<0.01
7 d	30.0 ± 5.3 Aa	56.7 ± 4.7 Aa	10.0 ± 1.7 Ab	45.6 ± 6.7 Aa	10.0 ± 2.4 Ab	12.6	<0.01
F	44.2	178.8	8.0	37.9	6.7		
P	<0.01	<0.01	<0.01	<0.01	<0.01		
	Concentration:	1000 ppm					
4 h	0.0 ± 0.0 D	0.0 ± 0.0 D	0.0 ± 0.0 C	0.0 ± 0.0 C	0.0 ± 0.0 B	-	-
8 h	0.0 ± 0.0 D	0.0 ± 0.0 D	0.0 ± 0.0 C	0.0 ± 0.0 C	0.0 ± 0.0 B	-	-
16 h	0.0 ± 0.0 D	0.0 ± 0.0 D	1.1 ± 1.1 C	0.0 ± 0.0 C	1.1 ± 1.1 AB	0.8	0.56
1 d	0.0 ± 0.0 Db	11.1 ± 2.6 Ca	7.8 ± 1.5 Ba	2.2 ± 1.5 Cb	2.2 ± 1.5 ABb	7.8	<0.01
2 d	11.1 ± 2.6 Cb	41.1 ± 3.5 Ba	17.8 ± 2.2 Aab	10.0 ±3.7 Bbc	2.2 ± 1.5 ABc	12.8	<0.01
3 d	27.8 ± 3.6 Bab	56.7 ± 5.2 ABa	25.6 ± 4.1 Ab	45.6 ± 6.5 Aab	2.2 ± 1.5 ABc	46.9	<0.01
4 d	42.2 ± 4.0 ABab	66.7 ± 6.2 ABa	26.7 ± 4.1 Ab	60.0 ± 5.0 Aab	5.6 ± 2.4 ABc	29.8	<0.01
5 d	54.4 ± 3.8 Aa	74.4 ± 6.0 ABa	30.0 ± 3.3 Aa	68.9 ± 5.4 Aa	7.8 ± 2.8 ABb	25.7	<0.01
6 d	58.9 ± 3.5 Aab	85.6 ± 5.6 Aa	34.4 ± 5.0 Ab	78.9 ±5.1 Aab	10.0 ± 2.9 Ac	22.9	<0.01
7 d	71.1 ± 3.1 Aa	90.0 ± 4.8 Aa	40.0 ± 5.3 Aa	80.0 ± 5.5 Aa	11.1 ± 3.1 Ab	21.7	<0.01
F	187.7	194.8	66.2	92.6	3.8		
P	<0.01	<0.01	<0.01	<0.01	<0.01		

For each EO, within each row, means followed by the same lowercase letter are not significantly different (df = 9, 89; Tukey HSD test at P = 0.05). For each concentration, within each column, means followed by the same uppercase letter are not significantly different (df = 4, 44; Tukey HSD test at P = 0.05). No significant differences were recorded where no letters exist. No statistical analysis was performed where dashes exist.

Table S7. Mean (%) mortality ± standard error (SE) of *Rhyzopertha dominica* adults after 4–16 h, and 1–7 days in wheat treated with *Citrus reticulata, Illicium verum, Monodora myristica,* and *Xylopia aethiopica* (under the abbreviations *C. r*, *I. v*, *M. m*, and *X. a*, respectively) EOs at two concentrations, and with positive control, pirimiphos-methyl (under the abbreviation P - m).

**EO type**	***C. r***	***I. v***	***M. m***	***X. a***	**P - m**		
			**Adults**				
**Exposure**	**Concentration:**	**500 ppm**				***F***	***P***
4 h	0.0 ± 0.0 C	0.0 ± 0.0 B	0.0 ± 0.0 D	0.0 ± 0.0 C	0.0 ± 0.0 D	-	-
8 h	0.0 ± 0.0 C	0.0 ± 0.0 B	0.0 ± 0.0 D	0.0 ± 0.0 C	2.2 ± 1.5 CD	2.3	0.08
16 h	0.0 ± 0.0 C	0.0 ± 0.0 B	0.0 ± 0.0 D	0.0 ± 0.0 C	2.2 ± 1.5 CD	2.3	0.08
1 d	0.0 ± 0.0 Cb	2.2 ± 1.5 Bab	0.0 ± 0.0 Db	0.0 ± 0.0 Cb	6.7 ± 2.4 Ca	5.8	<0.01
2 d	0.0 ± 0.0 Cc	8.9 ± 2.0 Ab	4.4 ± 1.8 Cb	0.0 ± 0.0 Cc	21.1 ± 2.0 Ba	24.6	<0.01
3 d	1.1 ± 1.1 BCc	12.2 ± 2.2 Aab	7.8 ± 1.5 BCb	3.3 ± 2.4 BCc	32.2 ± 2.2 ABa	18.7	<0.01
4 d	4.4 ± 2.9 ABCc	13.3 ± 2.9 Ab	11.1 ± 1.1 ABb	5.6 ± 3.4 BCc	44.4 ± 2.9 ABa	15.3	<0.01
5 d	8.9 ± 4.6 ABCbc	14.4 ± 2.4 Aab	12.2 ± 1.5 ABb	8.9 ± 4.8 ABc	55.6 ± 2.9 ABa	11.7	<0.01
6 d	10.0 ± 4.4 ABbc	15.6 ± 2.4 Ab	14.4 ± 1.8 ABb	11.1 ± 5.9 Ac	68.9 ± 3.1 Aa	11.4	<0.01
7 d	14.4 ± 5.3 Abc	15.6 ± 2.4 Ab	15.6 ± 1.8 Ab	13.3 ± 7.3 Ac	81.1± 2.6 Aa	10.4	<0.01
F	5.3	29.2	45.8	2.2	63.4		
P	<0.01	<0.01	<0.01	0.03	<0.01		
	Concentration:	1000 ppm					
4 h	0.0 ± 0.0 E	0.0 ± 0.0 B	0.0 ± 0.0 C	0.0 ± 0.0 E	0.0 ± 0.0 D	-	-
8 h	0.0 ± 0.0 E	0.0 ± 0.0 B	0.0 ± 0.0 C	0.0 ± 0.0 E	2.2 ± 1.5 CD	2.3	0.08
16 h	0.0 ± 0.0 Eb	0.0 ± 0.0 Bb	0.0 ± 0.0 Cb	0.0 ± 0.0 Eb	3.3 ± 1.7 CDa	4.0	<0.01
1 d	5.6 ± 1.8 Da	6.7 ± 1.7 ABa	0.0 ± 0.0 Cb	0.0 ± 0.0 Eb	5.6 ± 2.4 Cab	5.3	<0.01
2 d	10.0 ± 1.7 CDa	12.2 ± 2.8A a	11.1 ± 3.1 Ba	0.0 ± 0.0 Eb	16.7 ± 2.4 Ba	13.0	<0.01
3 d	17.8 ± 2.8 BCab	18.9 ± 4.2 Aab	16.7 ± 2.9 ABab	8.9 ± 2.6 Db	28.9 ± 3.1 ABa	3.7	0.01
4 d	24.4 ± 2.9 ABab	21.1 ± 5.1 Ab	16.7 ± 2.9 ABab	22.2 ± 2.2 Cab	40.0 ± 3.3 ABa	3.1	0.03
5 d	31.1 ± 4.6 ABab	21.1 ± 5.1 Ab	17.8 ± 2.8 Ab	31.1 ± 3.1 BCab	51.1 ± 4.2 Aa	4.5	<0.01
6 d	37.8 ± 4.7 ABab	21.1 ± 5.1 Ac	20.0 ± 2.4 Abc	48.9 ±3.5 ABab	67.8 ± 2.8 Aa	8.1	<0.01
7 d	46.7 ± 6.7 Aab	21.1 ± 5.1 Ac	20.0 ± 2.4 Abc	63.3 ± 4.1 Aa	81.1 ± 5.4 Aa	10.5	<0.01

F	68.2	9.0	77.4	145.2	52.1
P	<0.01	<0.01	<0.01	<0.01	<0.01

For each EO, within each row, means followed by the same lowercase letter are not significantly different (df = 9, 89; Tukey HSD test at P = 0.05). For each concentration, within each column, means followed by the same uppercase letter are not significantly different (df = 4, 44; Tukey HSD test at P = 0.05). No significant differences were recorded where no letters exist. No statistical analysis was performed where dashes exist.

Table S8. Mean (%) mortality ± standard error (SE) of *Sitophilus oryzae* adults after 4–16 h, and 1–7 days in wheat treated with *Citrus reticulata, Illicium verum, Monodora myristica,* and *Xylopia aethiopica* (under the abbreviations *C. r*, *I. v*, *M. m*, and *X. a*, respectively) EOs at two concentrations, and with positive control, pirimiphos-methyl (under the abbreviation P - m).

**EO type**	***C. r***	***I. v***	***M. m***	***X. a***	**P - m**		
			**Adults**				
**Exposure**	**Concentration:**	**500 ppm**				***F***	***P***
4 h	3.3 ± 1.7 Ea	0.0 ± 0.0 Bb	0.0 ± 0.0 Bb	0.0 ± 0.0 Bb	0.0 ± 0.0 Fb	4.0	<0.01
8 h	14.4 ± 2.4 Da	0.0 ± 0.0 Bb	0.0 ± 0.0 Bb	0.0 ± 0.0 Bb	0.0 ± 0.0 Fb	57.5	<0.01
16 h	22.2 ± 2.8 CDa	0.0 ± 0.0 Bb	0.0 ± 0.0 Bb	0.0 ± 0.0 Bb	0.0 ± 0.0 Fb	474.0	<0.01
1 d	36.7 ± 2.9 BCa	2.2 ± 1.5 ABc	0.0 ± 0.0 Bc	0.0 ± 0.0 Bc	8.9 ± 2.0 Eb	44.5	<0.01
2 d	50.0 ± 3.3 ABa	4.4 ± 1.8 ABc	0.0 ± 0.0 Bd	0.0 ± 0.0 Bd	21.1 ± 1.1 Db	87.7	<0.01
3 d	63.3 ± 4.1 ABa	7.8 ± 2.8 Ab	0.0 ± 0.0 Bc	4.4 ± 1.8 Abc	38.9 ± 2.0 Ca	38.2	<0.01
4 d	80.0 ± 2.4 ABa	7.8 ± 2.8 Ab	0.0 ± 0.0 Bc	4.4 ± 1.8 Abc	48.9 ± 3.5 BCa	43.9	<0.01
5 d	94.4 ± 1.8 Aa	7.8 ± 2.8 Ab	0.0 ± 0.0 Bc	4.4 ± 1.8 Abc	66.7 ± 4.1 ABCa	49.9	<0.01
6 d	98.9 ± 1.1 Aa	7.8 ± 2.8 Ab	1.1 ± 1.1 ABb	4.4 ± 1.8 Ab	75.6 ± 3.8 ABa	41.4	<0.01
7 d	100.0 ± 0.0 Aa	7.8 ± 2.8 Ab	3.3 ± 1.7 Ab	4.4 ± 1.8 Ab	92.2± 2.8 Aa	32.4	<0.01
F	48.3	3.3	2.9	3.6	237.3		
P	<0.01	<0.01	<0.01	<0.01	<0.01		
	Concentration:	1000 ppm					
4 h	3.3 ± 1.7 Da	0.0 ± 0.0 Db	0.0 ± 0.0 Cb	0.0 ± 0.0 Bb	0.0 ± 0.0 Fb	4.0	<0.01
8 h	18.9 ± 2.6 Ca	0.0 ± 0.0 Db	0.0 ± 0.0 Cb	0.0 ± 0.0 Bb	0.0 ± 0.0 Fb	434.7	<0.01
16 h	34.4 ± 4.8 BCa	2.2 ± 1.5 Db	0.0 ± 0.0 Cb	0.0 ± 0.0 Bb	0.0 ± 0.0 Fb	79.6	<0.01
1 d	53.3 ± 5.0 ABa	8.9 ± 1.1 Cb	0.0 ± 0.0 Cc	0.0 ± 0.0 Bc	10.0 ± 1.7 Eb	87.5	<0.01
2 d	72.2 ± 4.3 Aa	17.8 ± 2.8 BCb	0.0 ± 0.0 Cc	1.1 ± 1.1 ABc	25.6 ± 2.9 Db	167.3	<0.01
3 d	85.6 ± 4.8 Aa	27.8 ± 3.6 ABb	0.0 ± 0.0 Cd	6.7 ± 2.4 ABc	40.0 ± 1.7 Cab	72.8	0.01
4 d	95.6 ± 2.9 Aa	32.2 ± 4.0 ABb	2.2 ± 1.5 BCc	6.7 ± 2.4 ABc	48.9 ± 2.0 BCab	43.1	0.03
5 d	98.9 ± 1.1 Aa	41.1 ± 6.3 ABa	4.4 ± 1.8 ABCb	6.7 ± 2.4 ABb	64.4 ± 2.9 ABa	32.8	<0.01
6 d	98.9 ± 1.1 Aa	41.1 ± 6.3 ABa	7.8 ± 2.2 ABb	8.9 ±2.6 Ab	76.7 ± 4.4 ABa	24.5	<0.01
7 d	98.9 ± 1.1 Aa	44.4 ± 6.5 Aa	11.1 ± 3.1 Ab	8.9 ±2.6 Ab	91.1 ± 3.1 Aa	21.4	<0.01
F	66.7	74.3	8.0	5.9	364.3		

P	<0.01	<0.01	<0.01	<0.01	<0.01

For each EO, within each row, means followed by the same lowercase letter are not significantly different (df = 9, 89; Tukey HSD test at P = 0.05). For each concentration, within each column, means followed by the same uppercase letter are not significantly different (df = 4, 44; Tukey HSD test at P = 0.05). No significant differences were recorded where no letters exist.

Table S9. Mean (%) mortality ± standard error (SE) of *Acarus siro* nymphs and adults after 4–16 h, and 1–7 days in wheat treated with *Citrus reticulata, Illicium verum, Monodora myristica,* and *Xylopia aethiopica* (under the abbreviations *C. r* , *I. v*, *M. m*, and *X. a*, respectively) EOs at two concentrations, and with positive control, pirimiphos-methyl (under the abbreviation P - m).

**EO type**	***C. r***	***I. v***	***M. m***	***X. a***	**P - m**		
			**Nymphs**				
**Exposure**	**Concentration:**	**500 ppm**				***F***	***P***
4 h	0.0 ± 0.0 D	0.0 ± 0.0 D	0.0 ± 0.0 B	0.0 ± 0.0 C	0.0 ± 0.0 D	-	-
8 h	0.0 ± 0.0 D	0.0 ± 0.0 D	0.0 ± 0.0 B	0.0 ± 0.0 C	0.0 ± 0.0 D	-	-
16 h	0.0 ± 0.0 D	0.0 ± 0.0 D	0.0 ± 0.0 B	0.0 ± 0.0 C	0.0 ± 0.0 D	-	-
1 d	1.1 ± 1.1 D	1.1 ± 1.1 D	0.0 ± 0.0 B	1.1 ± 1.1 BC	1.1 ± 1.1 D	0.3	0.91
2 d	3.3 ± 1.7 CD	2.2 ± 1.5 D	0.0 ± 0.0 B	2.2 ± 4.4 BC	3.3 ± 1.7 D	0.9	0.45
3 d	5.6 ± 2.4 CDab	6.7 ± 3.3 CDab	1.1 ± 1.1 Bb	4.4 ± 1.8 Bab	8.9 ± 1.1 Ca	2.9	0.03
4 d	15.6 ± 4.1 BCab	20.0 ± 6.2 BCa	3.3 ± 1.7 ABb	13.3 ± 2.4 Aa	18.9 ± 2.6 BCa	4.3	<0.01
5 d	25.6 ± 5.0 ABa	28.9 ± 7.3 ABa	3.3 ± 1.7 ABb	17.8 ± 1.5 Aa	26.7 ± 3.7 ABa	12.2	<0.01
6 d	36.7 ± 5.0 Aa	44.4 ± 8.0 ABa	4.4 ± 1.8 ABb	21.1 ± 2.0 Aa	41.1 ± 3.9 ABa	22.4	<0.01
7 d	48.9 ± 5.9 Aa	58.9 ± 7.9 Aa	7.8 ± 1.5 Ab	28.9 ± 3.9 Aa	51.1 ± 3.9 Aa	22.1	<0.01
F	33.6	27.3	5.9	38.1	77.2		
P	<0.01	<0.01	<0.01	<0.01	<0.01		
	Concentration:	1000 ppm					
4 h	0.0 ± 0.0 D	0.0 ± 0.0 C	0.0 ± 0.0 C	0.0 ± 0.0 C	0.0 ± 0.0 E	-	-
8 h	0.0 ± 0.0 D	0.0 ± 0.0 C	0.0 ± 0.0 C	0.0 ± 0.0 C	0.0 ± 0.0 E	-	-
16 h	0.0 ± 0.0 D	0.0 ± 0.0 C	0.0 ± 0.0 C	0.0 ± 0.0 C	0.0 ± 0.0 E	-	-
1 d	2.2 ± 2.2 D	2.2 ± 1.5 C	0.0 ± 0.0 C	2.2 ± 1.5 C	1.1 ± 1.1 E	0.6	0.69
2 d	6.7 ± 2.9 CD	5.6 ± 2.9 BC	2.2 ± 1.5 C	4.4 ± 2.4 BC	2.2 ± 1.5 DE	0.4	0.76
3 d	11.1 ± 5.1 CD	11.1 ± 3.5 B	4.4 ± 2.4 BC	8.9 ± 2.6 B	6.7 ± 1.7 CD	0.7	0.61
4 d	23.3 ± 7.3 BCa	31.1 ± 5.6 Aa	4.4 ± 2.4 BCb	18.9 ± 2.6 Aa	14.4 ± 2.4 BCa	6.8	<0.01
5 d	35.6 ± 7.7 ABa	43.3 ± 7.3 Aa	6.7 ± 2.9 ABCb	28.9 ± 3.9 Aa	23.3 ± 3.7 ABa	9.5	<0.01
6 d	47.8 ± 8.0 ABa	53.3 ± 8.2 Aa	11.1 ± 2.0 ABb	33.3 ± 2.9 Aa	38.9 ± 3.1 Aa	13.4	<0.01
7 d	58.9 ± 7.7 Aa	65.6 ± 9.1 Aa	14.4 ± 2.4 Ab	40.0 ± 1.7 Aa	52.2 ± 3.2 Aa	14.3	<0.01

F	26.1	41.7	9.2	43.1	41.2		
P	<0.01	<0.01	<0.01	<0.01	<0.01		
			**Adults**				
	Concentration:	500 ppm					
4 h	0.0 ± 0.0 E	0.0 ± 0.0 E	0.0 ± 0.0 D	0.0 ± 0.0 E	0.0 ± 0.0 F	-	-
8 h	0.0 ± 0.0 E	0.0 ± 0.0 E	0.0 ± 0.0 D	0.0 ± 0.0 E	0.0 ± 0.0 F	-	-
16 h	0.0 ± 0.0 E	0.0 ± 0.0 E	0.0 ± 0.0 D	0.0 ± 0.0 E	1.1 ± 1.1 F	1.0	0.42
1 d	2.2 ± 1.5 E	3.3 ± 1.7 E	0.0 ± 0.0 D	2.2 ± 1.5 E	3.3 ± 1.7 EF	0.9	0.45
2 d	5.6 ± 1.8 DEabc	8.9 ± 3.1 DEab	1.1 ± 1.1 Dc	3.3 ± 1.7 Ebc	13.3 ± 1.7 Ea	6.5	<0.01
3 d	10.0 ± 2.4 DEab	17.8 ± 4.9 CDEab	2.2 ± 1.5 CDc	7.8 ± 2.2 DEbc	25.6 ± 2.4 Da	8.6	<0.01
4 d	20.0 ± 4.1 CDa	31.1 ± 6.6 BCDa	5.6 ± 1.8 BCDb	18.9 ± 4.6 CDa	41.1 ± 3.1 Ca	7.3	<0.01
5 d	31.1 ± 4.6 BCa	44.4 ± 7.8 ABCa	8.9 ± 2.6 ABCb	24.4 ± 4.1 BCa	56.7 ± 4.4 Ba	7.9	<0.01
6 d	45.6 ± 6.9 ABa	55.6 ± 9.0 ABa	10.0 ± 2.4 ABb	36.7 ± 6.0 ABa	64.4 ± 2.9 ABa	12.6	<0.01
7 d	56.7 ± 6.0 Aa	68.9 ± 10.7 Aa	13.3 ± 1.7 Ab	45.6 ± 4.4 Aa	71.1 ± 2.6 Aa	21.1	<0.01
F	38.7	28.2	13.3	42.0	127.9		
P	<0.01	<0.01	<0.01	<0.01	<0.01		
	Concentration:	1000 ppm					
4 h	0.0 ± 0.0 D	0.0 ± 0.0 E	0.0 ± 0.0 E	0.0 ± 0.0 E	0.0 ± 0.0 E	-	-
8 h	0.0 ± 0.0 D	0.0 ± 0.0 E	0.0 ± 0.0 E	0.0 ± 0.0 E	0.0 ± 0.0 E	-	-
16 h	0.0 ± 0.0 D	0.0 ± 0.0 E	0.0 ± 0.0 E	0.0 ± 0.0 E	2.2 ± 1.5 DE	2.3	0.08
1 d	3.3 ± 1.7 CD	4.4 ± 1.8 D	1.1 ± 1.1 DE	2.2 ± 1.5 DE	4.4 ± 1.8 D	0.9	0.50
2 d	8.9 ± 2.6 BCab	11.1 ± 3.1 CDab	3.3 ± 1.7 CDEb	5.6 ± 2.4 CDab	14.4 ± 1.8 Ca	3.7	0.01
3 d	14.4 ± 4.4 Bab	23.3 ± 4.4 BCa	5.6 ± 1.8 BCDb	12.2 ± 2.8 BCab	26.7 ± 2.9 BCa	5.1	<0.01
4 d	26.7 ± 6.7 ABab	35.6 ± 5.6 ABa	10.0 ± 2.4 ABCb	26.7 ± 4.1 ABa	40.0 ± 3.7 ABa	5.2	<0.01
5 d	38.9 ± 6.8 Aa	51.1 ± 5.9 ABa	13.3 ± 2.9 ABb	28.9 ± 4.6 Aa	51.1 ± 3.9 ABa	10.0	<0.01
6 d	53.3 ± 8.3 Aa	64.4 ± 9.2 Aa	17.8 ± 2.2 Ab	45.6 ± 4.8 Aa	63.3 ± 4.1 Aa	17.2	<0.01
7 d	66.7 ± 7.6 Aa	75.6 ± 6.7 Aa	21.1 ± 1.1 Ab	55.6 ± 4.8 Aa	72.2 ± 2.8 Aa	39.1	<0.01
F	35.4	66.8	19.5	51.9			
P	<0.01	<0.01	<0.01	<0.01	<0.01		

For each EO, within each row, means followed by the same lowercase letter are not significantly different (df = 9, 89; Tukey HSD test at P = 0.05). For each concentration, within each column, means followed by the same uppercase letter are not significantly different (df = 4, 44; Tukey HSD test at P = 0.05). No significant differences were recorded where no letters exist. No statistical analysis was performed where dashes exist.
